# Virtual cells: progress and challenges from mechanistic modeling to data-driven integration

**DOI:** 10.1093/bib/bbag562

**Published:** 2026-10-18

**Authors:** Nuoran Ma, Zihao Wang, Xu Yang, Guoxing Yang, Rui Yang, Zhenxing Wang, Qinghua Jiang, Yanshuo Chu

**Affiliations:** Department of Artificial Intelligence, School of Future Technology, Harbin Institute of Technology, 92 Xidazhi Street, Nangang District, Harbin 150001, Heilongjiang, China; Harbin Institute of Technology, Harbin 150001, Heilongjiang, China; Harbin Institute of Technology, Harbin 150001, Heilongjiang, China; Harbin Institute of Technology, Harbin 150001, Heilongjiang, China; Harbin Institute of Technology, Harbin 150001, Heilongjiang, China; School of Computer Science and Artificial Intelligence, Northeast Forestry University, Harbin 150040, Heilongjiang, China; Center for Bioinformatics, School of Life Science and Technology, Harbin Institute of Technology, Harbin 150001, Heilongjiang, China; School of Medicine and Health, Harbin Institute of Technology, Harbin 150000, Heilongjiang, China

**Keywords:** virtual cell, single-cell foundation model, perturbation prediction, multimodal omics, spatial and multiscale modeling, experimental validation

## Abstract

Virtual cells aim to represent cellular states computationally and predict responses to genetic, chemical, environmental, temporal, and spatial conditions. For comparative purposes, this review uses state encoding (E), state transition (T), and observation decoding (D) as a conceptual abstraction spanning explicit mechanistic models, data-driven foundation and perturbation models, multimodal systems, and spatial and multiscale simulation. We examine model boundaries, data foundations, resource disclosure, out-of-distribution evaluation, causal evidence, and experimental validation. Independent benchmarks show that parameter count and training-set size do not automatically yield robust out-of-distribution generalization, while transcriptomic representations alone do not capture the full functional state of a cell. Trustworthy virtual cells will require perturbation-responsive transitions, cross-modal alignment, continuous-time dynamics, mechanistic constraints, calibrated uncertainty, independent experimental validation, and, where appropriate, closed-loop experimental feedback.

## Introduction

The central problem for virtual cells is how to describe cellular state computationally and predict changes in response to genetic, chemical, environmental, and spatial signals. Traditional models derive dynamical equations from biochemical reactions, molecular diffusion, membrane potentials, and cellular mechanics, with explicit variables and parameters. Data-driven models instead learn statistical structure from large-scale single-cell data to predict unobserved states. These approaches are complementary: one emphasizes mechanistic prior knowledge and the other patterns learned from data.

A single-cell foundation model does not automatically become a complete virtual cell because it has many parameters or general representational capacity. A model used only for cell-type annotation, dimensionality reduction, or batch correction is more accurately described as a cell-representation model. In the operational framework adopted in this review, large cellular models (LCMs) primarily emphasize transferable state representations, whereas artificial intelligence virtual cells (AIVCs) place additional emphasis on perturbation-responsive state transitions, conditional or temporal context, and experimentally testable outputs [1]. This LCM/AIVC distinction is an analytical convention used for comparison in this review rather than a field-wide standardized taxonomy. Their long-term goal is to connect molecular modalities, cellular states, tissue environments, and temporal scales so that researchers can perturb cells *in silico*, observe responses, and propose testable hypotheses.

For comparative purposes, virtual-cell computation can be abstracted into state encoding, state transition, and observation decoding. E/T/D is therefore a conceptual comparative abstraction rather than a mandatory architectural decomposition. Let $X_{t}$ denote a single-cell state vector or population expression matrix at time *t*. Let $M_{t}$ denote other molecular and imaging modalities, including proteins, chromatin, morphology, and ultrastructure. Let $S_{t}$ denote spatial context, *P* a genetic, chemical, or environmental perturbation, and *C* condition variables such as cell type, tissue of origin, individual, dose, and experimental batch. The state encoder *E* maps observations to a latent state:


(1) \begin{eqnarray*}& z_{t} = E\left( X_{t},M_{t},S_{t},C \right).\end{eqnarray*}

For tasks without spatial information, $S_{t}$ may be omitted. The state-transition operator *T* predicts changes in the latent state from the perturbation, spatial environment, and time interval:


(2) \begin{eqnarray*}& z_{t + \Delta t} = T\left( z_{t},P,S_{t},C,\Delta t \right).\end{eqnarray*}

Let $Y_{t + \Delta t}$ denote the target observation. It may comprise one or more measurable modalities, including gene expression, protein abundance, cell morphology, or ultrastructure. The observation decoder *D* converts the latent state into the target observation:


(3) \begin{eqnarray*}& {\widehat{Y}}_{t + \Delta t} = D\left( z_{t + \Delta t} \right).\end{eqnarray*}

Because cellular heterogeneity and biological stochasticity generate multiple possible outcomes, the objective is to learn a conditional distribution of future observations:


(4) \begin{eqnarray*}& p\left( Y_{t + \Delta t} \mid X_{t},M_{t},S_{t},P,C,\Delta t \right).\end{eqnarray*}

Let $O_{t} = \left ( X_{t},M_{t},S_{t},C \right )$ denote the joint observation and let *F* denote the overall predictive mapping. The objective can then be written as


(5) \begin{eqnarray*}& F\left( O_{t},P,\Delta t \right) = p\left( Y_{t + \Delta t} \mid O_{t},P,\Delta t \right).\end{eqnarray*}

The main difference among approaches is how E-like representation, T-like state evolution, and D-like output functions are implemented or expressed. In learned systems these roles may correspond to separable modules, but in mechanistic systems they are usually coupled. In Virtual Cell, experimentally specified molecular and cellular state variables serve an E-like representational role; the governing ordinary differential equations (ODEs), partial differential equations (PDEs), and stochastic dynamics provide the T-like state-evolution role; and simulated observables provide the D-like output role [2]. In PhysiCell, agent phenotype and microenvironment variables, rule/force/event dynamics, and simulated population or tissue outputs play analogous functional roles [3]. These roles are conceptually mapped rather than implemented as three mandatory independent modules. System-level virtual cells therefore need to connect these functional roles while maintaining biological consistency across scales.

The early Virtual Cell platform converted experimentally obtained biochemical, electrophysiological, and imaging information into ODEs, PDEs, and stochastic models, providing a reproducible simulation environment for intracellular signal transduction, molecular diffusion, and membrane transport [2]. Whole-cell models developed by Karr *et al*. [4] further integrated multiple submodels of cellular processes, attempting to predict cellular phenotypes from genotypes. Agent-based simulation frameworks such as PhysiCell represent individual cells as computational agents with volume, cell-cycle, death, motility, and mechanical properties, enabling models to study emergent behaviors in cell populations and tissue microenvironments [3].

Because transcriptomics captures only one layer of cellular state, virtual-cell models increasingly incorporate chromatin, protein, spatial, imaging, and ultrastructural information. Representative directions include EpiAgent for chromatin accessibility, Nicheformer and SToFM for spatial context, CAPTAIN and HoloCell for multimodal state representation, ProteinTalks for protein-network dynamics, and EM-based models for subcellular structure [5–14]. These extensions broaden the E/T/D abstraction beyond a single expression vector.

Large-scale pretraining and perturbation models likewise shift the field from manually specified kinetics toward learned representations and intervention-responsive states [15–24]. However, independent benchmarks show that greater scale or complexity does not consistently improve zero-shot or out-of-distribution (OOD) perturbation prediction [25, 26]. Figure 1 summarizes this historical progression.

**Figure 1:**
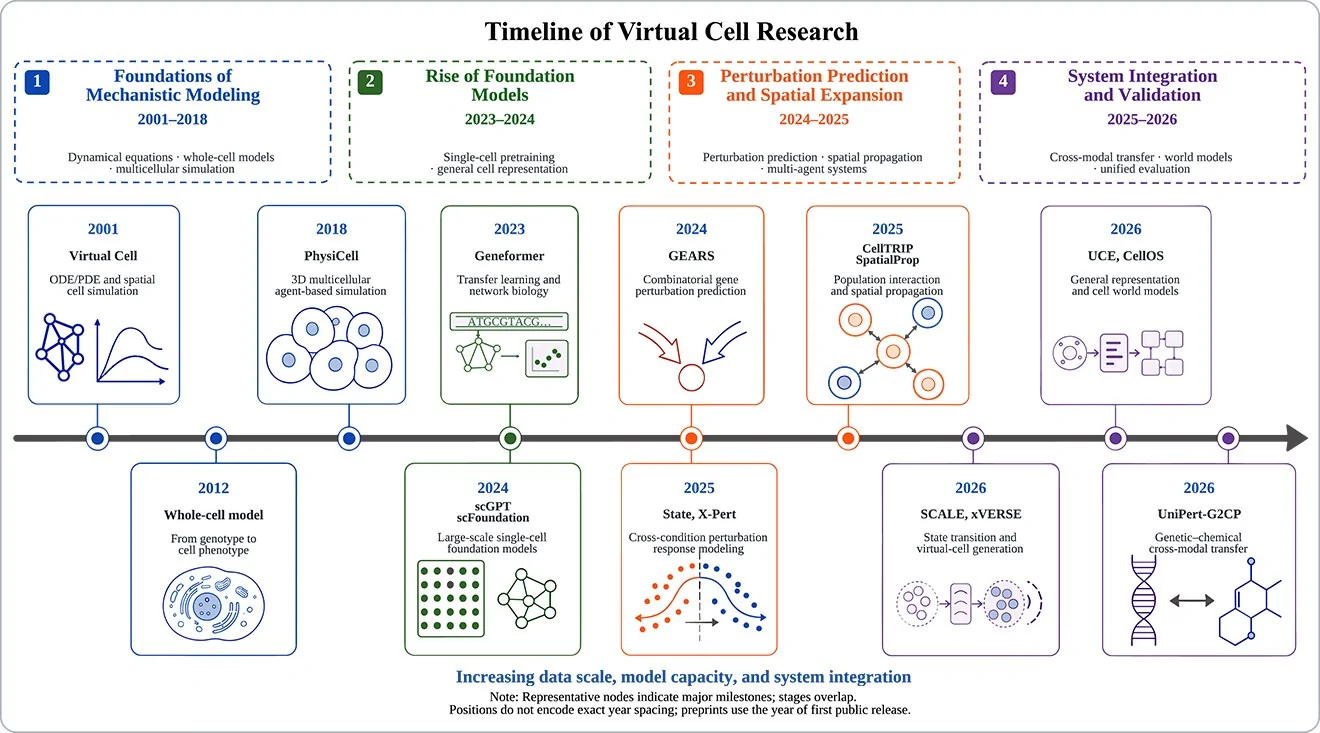
Timeline of virtual cell research. The nodes summarize technological development and are not positioned in strict proportion to calendar time.

Figure 2 summarizes the computational workflow from multisource data and prior knowledge through foundational representation, structured state transitions, spatial and multiscale simulation, and independent validation and updating. The review follows a conceptual progression from explicit mechanistic modeling to data-driven and multiscale systems and therefore does not mirror the workflow order shown in Fig. 2. The subsequent sections address shared data, reproducibility, evaluation, and experimental-update requirements. Figure 1 emphasizes historical evolution, whereas Fig. 2 positions major approaches within the computational process.

**Figure 2:**
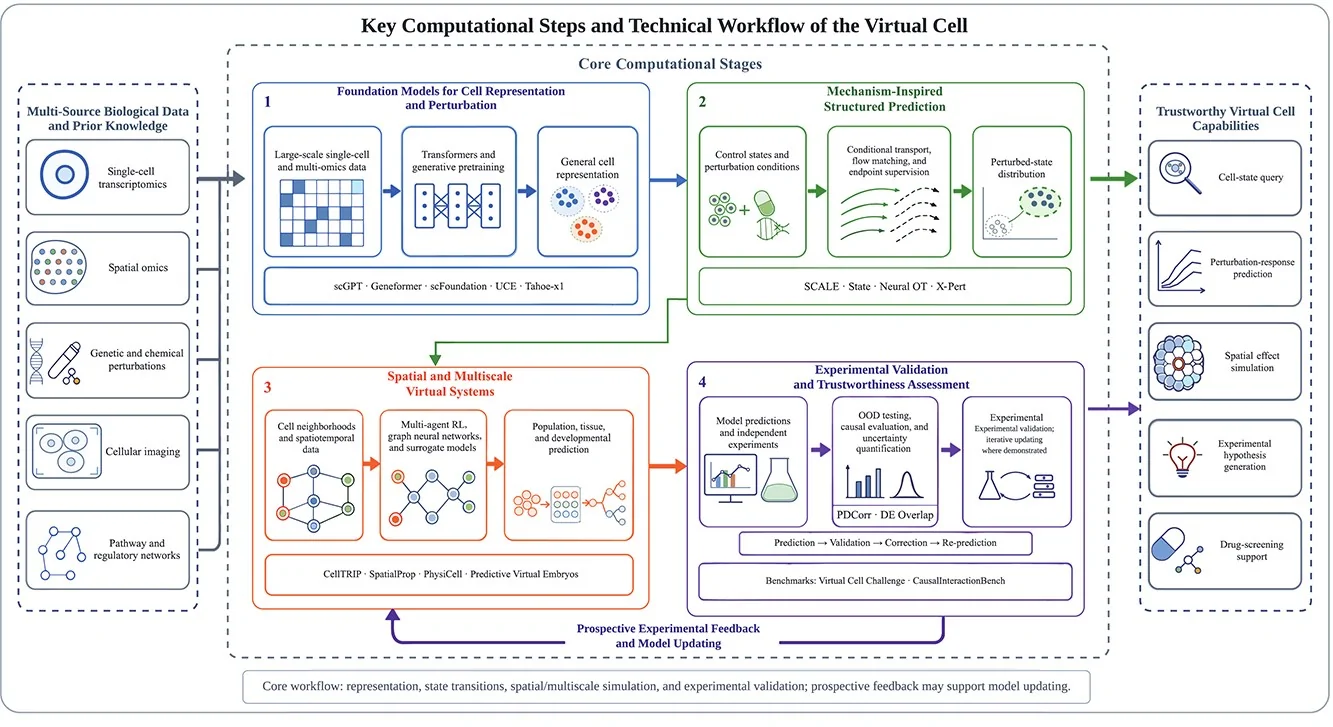
Key computational steps and technical workflow of the virtual cell. The final stage emphasizes experimental validation and trustworthiness assessment, while prospective experimental feedback may support iterative model updating where demonstrated. PDCorr and DE Overlap assess predictive agreement; they do not by themselves provide causal evidence and are not model-training constraints. Abbreviations: DE, differential expression; OT, optimal transport; PDCorr, Pearson Delta Correlation; RL, reinforcement learning.

E, T, and D provide a conceptual comparative abstraction, which Fig. 2 operationalizes as a four-stage workflow. Table 1 presents the operational LCM/AIVC distinction used in this review, whereas Fig. 3 and Table 2 support taxonomy and model retrieval. Five complementary evidence dimensions are also considered: documentation, task-specific empirical evidence, external and OOD generalization, mechanistic or causal evidence, and independent experimental validation. Closed-loop experimental feedback is reported separately from these dimensions.

**Table 1:** Operational comparison of LCMs and AIVCs used in this review.

Comparison dimension	Large cellular models	AI virtual cells
Primary objective	Emphasizes transferable cell and gene representations for annotation, integration, generation, and downstream adaptation	Emphasizes querying, prediction, or simulation of state changes under specified cellular contexts and intervention conditions
Primary inputs	Mainly large-scale scRNA-seq, with gradual extension to multi-omics	Transcriptomics, epigenomics, proteomics, spatial data, imaging, ultrastructure, and perturbation conditions
State representation	Typically emphasizes general embeddings and pretrained encoders that can be reused	Typically seeks to connect molecular, cellular, spatial, or temporal states while preserving conditional dependence
Core computation	Often emphasizes tokenization, embedding, pretraining, zero-shot inference, or fine-tuning, with a strong E-like representational role	May connect E-like representation, T-like state evolution, and D-like output/prediction functions; these need not be implemented as three separate learned modules
Perturbation and time	May be absent or handled only in downstream adaptation; many uses remain based on static representations	Usually places greater emphasis on perturbation-responsive, conditional, or temporal state evolution
Output capability	Annotation, retrieval, integration, embedding, or conditional generation	State querying, perturbation prediction, cross-modal generation, multiscale simulation, and hypothesis generation
Key validation	Downstream-task performance, transfer, and where relevant zero-shot evaluation	Task performance plus explicit generalization, mechanistic/causal, and experimental evidence as relevant to the reported system
Scope	Appropriate for representation learning, data integration, and downstream transfer; scale alone does not make a model a virtual cell	Appropriate for simulating state transitions under perturbations and supporting experimental decision-making; conclusions remain bounded by the training domain, mechanistic assumptions, and validation level

Note. The LCM/AIVC distinction is an operational analytical convention adopted in this review, not a field-wide standardized taxonomy. E/T/D describes functional emphasis rather than a mandatory architectural decomposition.

**Table 2:** Curated representative landscape of virtual-cell and related models from 2022 to 2026.

Model	Year	Primary modality	Core positioning and capabilities	Publication form	Reference
**Mechanistic and structured prediction**					
SCALE	2026	Perturbation scRNA-seq	Conditional flow matching that maps a control population to a perturbed-state distribution	Preprint	[20]
RegFormer	2026	scRNA-seq + gene regulatory networks	GRN hierarchy and state-space modeling; structural priors for representation and perturbation modeling	Peer-reviewed journal article	[7]
**Transcriptomic foundation models**					
scBERT	2022	scRNA-seq	BERT-style expression encoding; an early single-cell language-model approach	Peer-reviewed journal article	[27]
Geneformer	2023	scRNA-seq	Expression-ranked gene-sequence pretraining; network biology and transfer learning	Peer-reviewed journal article	[16]
CellPLM	2024	scRNA-seq + spatial transcriptomics	Cell latent representations, intercellular relationships, and multitask transfer	Peer-reviewed conference paper	[28]
scGPT	2024	scRNA-seq + multi-omics	Generative pretraining with adaptation to annotation, integration, perturbation, and other tasks	Peer-reviewed journal article	[15]
scFoundation	2024	scRNA-seq	Large-scale expression-value modeling for cell and gene representations and downstream transfer	Peer-reviewed journal article	[17]
GeneCompass	2024	scRNA-seq + prior knowledge	Cross-species pretraining that integrates regulatory knowledge	Peer-reviewed journal article	[29]
scMulan	2024	scRNA-seq + metadata	Multitask generative pretraining for annotation, batch integration, and conditional cell generation	Peer-reviewed conference paper	[30]
Cell2Sentence	2024	scRNA-seq + text	Converts cells into gene sentences to connect single-cell data with general language models	Peer-reviewed conference paper	[31]
xVERSE	2026	scRNA-seq + spatial transcriptomics	Transcriptomics-native probabilistic generation and general cell representation	Preprint	[32]
UCE	2026	scRNA-seq + protein sequences	Protein-language-model encoding for unified cell embeddings across tissues and species	Peer-reviewed journal article	[18]
TranscriptFormer	2026	scRNA-seq	Generative cross-species cell atlas and evolutionary-scale representations	Peer-reviewed journal article	[19]
**Epigenomics and unified multi-omics**					
scMVP	2022	RNA + ATAC	Joint generative modeling and latent representations for paired multi-omics	Peer-reviewed journal article	[33]
scCLIP	2023	RNA + ATAC	Dual-encoder contrastive learning and cross-modal alignment	Workshop paper	[34]
scmFormer	2024	RNA + protein	Multitask Transformer integration and generation of unmeasured modalities	Peer-reviewed journal article	[35]
EpiFoundation	2025	scATAC-seq + RNA supervision	Peak-to-gene alignment for annotation, batch correction, and expression prediction	Preprint	[36]
EpiAgent	2025	scATAC-seq	Large-scale chromatin-accessibility foundation model for perturbation prediction and cCRE virtual knockout	Peer-reviewed journal article	[5]
CellHermes	2025	Transcriptome + protein-interaction network + text	Converts heterogeneous omics into question answering to unify encoding, prediction, and interpretation	Preprint	[37]
CAPTAIN	2026	Paired RNA + surface protein	Joint RNA-protein representation for protein inference, annotation, and multi-omics integration	Peer-reviewed journal article	[14]
CLM-X	2026	RNA + ATAC	Unified processing of unpaired and paired inputs for cross-modal translation and perturbation prediction	Preprint	[38]
HoloCell	2026	Epigenome + transcriptome + proteome	Joint representation, alignment, and cross-modal generation across three omics layers	Preprint	[8]
SCARF	2025	RNA + ATAC	Mamba-based modality-specific and shared representations for annotation, matching, and cross-omics prediction	Preprint	[39]
**Perturbation and dynamic models**					
CPA	2023	scRNA-seq + drugs and dose	Disentangles cellular background and perturbation representations to predict complex intervention responses	Peer-reviewed journal article	[40]
Neural OT	2023	scRNA-seq	Neural optimal transport for population-level perturbation responses	Peer-reviewed journal article	[41]
GEARS	2024	CRISPR Perturb-seq	Graph priors and deep learning for multigene combinatorial perturbation prediction	Peer-reviewed journal article	[42]
State	2026	Large-scale perturbation transcriptomics	Perturbation-response modeling across cellular contexts	Peer-reviewed journal article	[21]
Stack	2026	scRNA-seq + conditioned cell sets	Tabular Transformer and gene-module tokens for in-context cross-condition prediction	Preprint	[43]
X-Pert	2025	Genetic + chemical perturbations	Unified modeling of perturbation types, combinations, and dose responses	Preprint	[22]
Tahoe-x1	2025	Drug-perturbation scRNA-seq	Billion-parameter joint representations of genes, cells, and compounds	Preprint	[23]
AlphaCell	2026	Whole-transcriptome states + perturbations	Conditional flow matching for continuous state transition, emphasizing unseen contexts	Preprint	[44]
X-Cell	2026	scRNA-seq + multimodal priors	Diffusion language model for genetic and drug perturbations with multimodal priors	Preprint	[24]
UniPert-G2CP	2026	Genetic + chemical perturbations	Transfers phenotype knowledge from genetic screens to chemical screens	Peer-reviewed journal article	[45]
SequenTx	2026	Tumor transcriptomics + drugs	Virtual-cell-inspired reinforcement learning for sequential drug design with *in vitro* and *in vivo* validation	Peer-reviewed journal article	[46]
**Spatial and multicellular systems**					
NicheCompass	2025	Spatial multi-omics + communication priors	Spatial-niche representation, communication programs, and reference mapping	Peer-reviewed journal article	[47]
SToFM	2025	Spatial transcriptomics	Multiscale pretraining across tissue macrostructure, microenvironment, and genes	Peer-reviewed conference paper	[10]
Nicheformer	2025	scRNA-seq + spatial omics	Pretraining on large-scale dissociated and spatial corpora to learn spatial context	Peer-reviewed journal article	[6]
STARNet	2025	Spatial RNA + ATAC	Heterogeneous hypergraph learning of spatially specific gene regulatory networks	Preprint	[48]
SpatialProp	2025	Spatial single-cell transcriptomics	Local perturbation propagation and directional-interaction evaluation on tissue graphs	Preprint	[49]
SpaRx	2023	Spatial transcriptomics + pharmacogenomics	Graph-based domain adaptation for spatially resolved single-cell drug-response prediction	Peer-reviewed journal article	[50]
CellTRIP	2025	Spatiotemporal single-cell states	Multi-agent reinforcement learning for cell-population interactions	Preprint	[51]
Predictive virtual embryos	2026	Single-cell + spatial + lineage	System-level research vision linking cell state, lineage, morphology, and development	Comment	[52]
VirTues	2026	Spatial proteomics	Multiscale protein, cell, niche, and tissue representation with cross-panel transfer and patient stratification	Peer-reviewed journal article	[53]
				**Proteomic virtual cells**	
CAPTAIN	2026	Paired RNA + surface protein	Protein-aware joint cell representation, emphasizing the functional execution layer	Peer-reviewed journal article	[14]
ProteinTalks	2026	Perturbation proteomics	Neural ODE for dynamic protein networks, drug efficacy, synergy, and resistance-related inference	Peer-reviewed journal article	[9]
VirTues	2026	Spatial proteomics	Hierarchical spatial-protein representation from molecular markers to tissue structure	Peer-reviewed journal article	[53]
**Electron microscopy and subcellular ultrastructure**					
EM-DINO and OmniEM	2025	2D and 3D EM	Large-scale pretraining for restoration, organelle segmentation, and high-resolution analysis	Preprint	[11]
EMCFsys	2025	Multimodal EM + vEM	Restoration, classification, semantic and instance segmentation, and sparsely labeled 3D reconstruction	Preprint	[12]
DF5T	2026	vEM + organelle EM	Denoising, deblurring, super-resolution, inpainting, and 3D isotropic restoration	Preprint	[13]
				**System-level models and intervention design**	
Virtual yeast	2026	Mechanistic knowledge + ultrastructure + dynamic omics	Systems-engineering vision for multifunctional modules and an active experimental closed loop	Perspective	[54]
GEMGen	2026	Perturbation transcriptomics + molecules	Reverse generation of candidate small molecules from target cellular phenotypes	Preprint	[55]
CellOS	2026	Multiview cell states	Joint embedding prediction for state consistency and world-model exploration	Preprint	[56]

Note. Years refer to the version of record when available; otherwise, the year of the first public preprint release is used. Publication form is reported separately from validation evidence. Table 2 is a curated representative landscape based on the revision-stage retrospective category-specific audit, not an exhaustive inventory. A model may appear under more than one technical category to indicate multi-category relevance and should not be interpreted as multiple distinct models. Abbreviations: ATAC, assay for transposase-accessible chromatin; BERT, bidirectional encoder representations from transformers; cCRE, candidate cis-regulatory element; CRISPR, clustered regularly interspaced short palindromic repeats; EM, electron microscopy; GRN, gene regulatory network; OT, optimal transport; RNA, ribonucleic acid; scATAC-seq, single-cell assay for transposase-accessible chromatin sequencing; scRNA-seq, single-cell RNA sequencing; vEM, volume electron microscopy.

**Figure 3:**
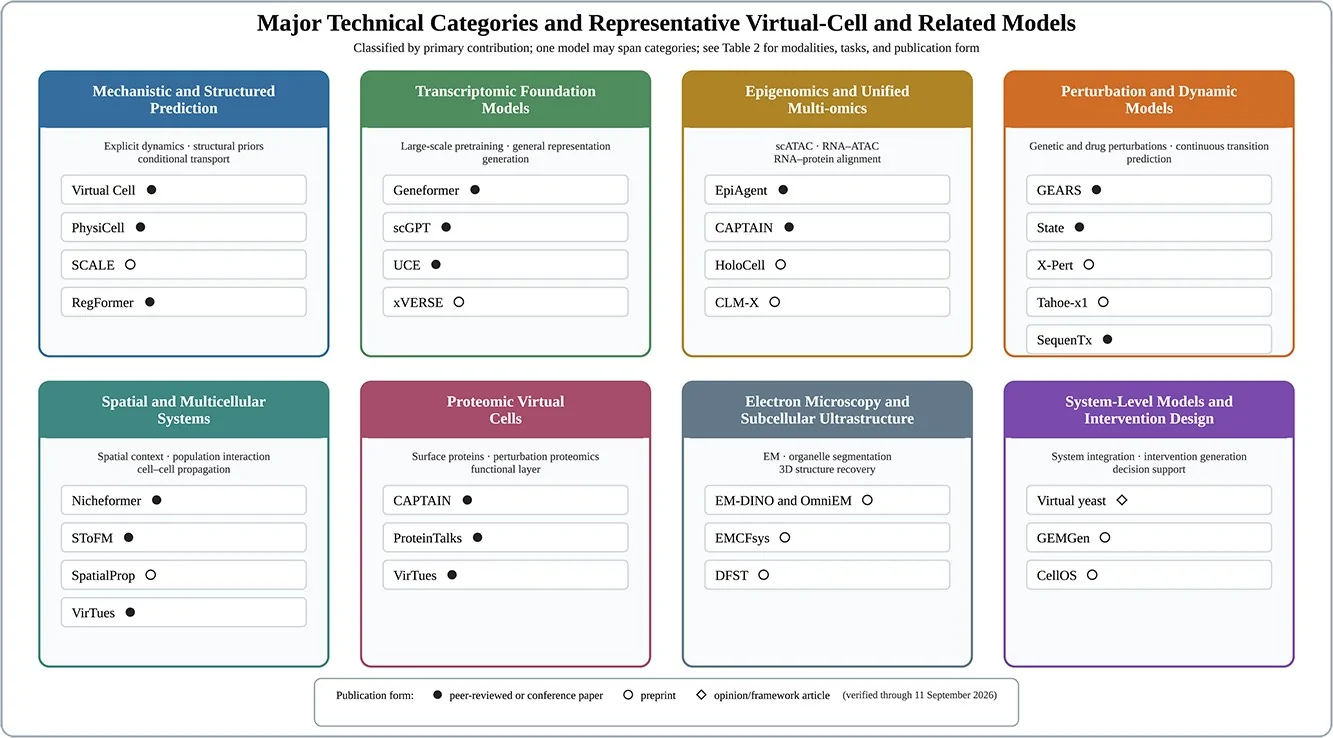
Major technical categories and representative virtual-cell and related models. Publication form was reverified through 11 September 2026. Model-reference mapping: Virtual Cell [2]; PhysiCell [3]; SCALE [20]; RegFormer [7]; Geneformer [16]; scGPT [15]; UCE [18]; xVERSE [32]; EpiAgent [5]; CAPTAIN [14]; HoloCell [8]; CLM-X [38]; GEARS [42]; State [21]; X-Pert [22]; Tahoe-x1 [23]; SequenTx [46]; Nicheformer [6]; SToFM [10]; SpatialProp [49]; VirTues [53]; ProteinTalks [9]; EM-DINO and OmniEM [11]; EMCFsys [12]; DF5T [13]; Virtual Yeast [54]; GEMGen [55]; CellOS [56]. Abbreviations: ATAC, assay for transposase-accessible chromatin; RNA, ribonucleic acid; scATAC, single-cell assay for transposase-accessible chromatin; vEM, volume electron microscopy.

### Literature scope and resource verification methods

This review combines narrative synthesis with structured literature and resource verification using a representative inclusion strategy. It remains a structured narrative review rather than a systematic review and does not claim exhaustive coverage, estimate publication bias, or perform quantitative synthesis. Historical works from 2001 onward trace virtual-cell development; models made public from 2022 through the revision-stage update construct the curated representative landscape in Table 2. Years refer to the version of record when available; otherwise, the year of the first public preprint release is used.

The retained submission records document broad source types, subject areas, and prioritization principles but do not contain a complete log of the original pre-submission searches. During revision, explicit category-specific inclusion criteria were therefore applied consistently to the existing Table 2 landscape, reviewer-suggested SpaRx, and other borderline cases in a retrospective verification and curation audit performed during revision. These revision-stage criteria are not represented as having been predefined before the initial submission. Table 2 is consequently a curated representative landscape rather than an exhaustive inventory. Borderline or cross-category methods are handled according to their primary technical contribution and direct relevance to virtual-cell development. Publication form is recorded separately from validation evidence.

Detailed information on the original records that can be confirmed, the revision-stage search/update process, representative search strings, category-specific inclusion and exclusion criteria, and borderline-case handling are provided in Supplementary Table S1. Supplementary Table S2 provides the model-level curation, role, capability, and evidence audit. Supplementary Table S3 records dated resource verification, including release, revision, model-card, and licensing information where ascertainable. Supplementary Table S4 records dataset provenance and overlap relevant to training/evaluation independence. Recent preprints are retained to capture emerging directions, but their capability claims remain provisional until peer review or independent replication where applicable.

## From explicit mechanistic simulation to mechanism-inspired structured prediction

### Explicit mechanistic models

Explicit mechanistic models define state variables, parameters, and transitions from known processes. Gene transcription, protein degradation, ligand–receptor binding, and metabolism can be represented by ODEs; intracellular diffusion and membrane transport by PDEs; and low-copy-number reactions by stochastic simulation. A general form is


(6) \begin{eqnarray*}& \frac{dx}{dt} = f(x,\theta,u,t).\end{eqnarray*}

Here, *x* denotes molecular concentration or cellular state, *θ* denotes parameters such as reaction rates, *u* denotes an external stimulus or control variable, and *t* denotes time. With spatial diffusion, the model becomes


(7) \begin{eqnarray*}& \frac{\partial x}{\partial t} = \kappa\nabla^{2}x + f(x,\theta,u,t).\end{eqnarray*}

Here, *κ* is the diffusion coefficient.

Virtual Cell translates experimentally defined cellular geometries, reaction networks, and boundary conditions into solvable mathematical models [2]. Explicit variables and parameters support interpretation, comparison of mechanistic hypotheses, and targeted experiments. However, large gene, protein, and spatial networks require extensive parameter estimation, and multiple timescales and high-dimensional nonlinearities complicate numerical solution.

Whole-cell models extend beyond local pathways. Karr *et al*. [4] integrated DNA replication, transcription, protein synthesis, metabolism, and cell-cycle submodels to simulate the life cycle of Mycoplasma genitalium. The study demonstrated coordination under a unified cellular state but also the dependence on manual curation, parameter estimation, and module interfaces. PhysiCell instead represents each cell as an agent governed by mechanics, movement, division, death, and phenotypic transitions, coupled to diffusion of nutrients, drugs, and signals [3]. This supports tumor, immune-migration, and morphogenesis simulations but reduces intracellular molecular state to relatively few phenotypic variables.

Between early pathway simulators and current foundation models, systems biology expanded through rule-based biochemical modeling, multicellular cell-based frameworks, reaction–diffusion coupling, and multiscale tissue simulation [57–59]. These approaches improved compositionality, spatial representation, and collective-cell simulation, but remained limited by parameterization burden, incomplete mechanistic knowledge, and computational scaling. Large single-cell atlases and standardized perturbation datasets later shifted part of the bottleneck from manually specifying every interaction toward learning transferable state representations and response maps. Foundation and perturbation models are therefore better viewed as a data-driven continuation of this history than as a discontinuous replacement of mechanistic systems biology.

### Mechanistic constraints, structured prediction, and biology-guided evaluation

Mechanistic constraints should be distinguished from biology-guided evaluation in data-driven virtual-cell research. Mechanistic constraints directly affect model structure or optimization, e.g. through reaction-network topology, conservation relations, or dynamical equations. PDCorr, DE Overlap, and pathway-enrichment consistency are biology-guided evaluation metrics when used only to compare predictions; they are not training constraints.

SCALE is discussed here only as a boundary case between structured prediction and mechanistic modeling; its perturbation-prediction architecture is reviewed in the subsection on perturbation-response prediction. Public materials describe PDCorr and DE Overlap as evaluation metrics rather than training constraints [20]. Accordingly, SCALE is treated as a structured, data-driven state-transition model rather than a traditional mechanistic dynamical model.

RegFormer introduces structural priors through gene-regulatory-network hierarchies and uses state-space models for long gene sequences [7]. Such priors constrain dependencies in the representation space without fully specifying the dynamics. Current approaches therefore form a continuum from explicit ODE, PDE, and stochastic models to probabilistic generative and large-scale pretrained models. A mechanistic label is justified only when biological knowledge constrains state variables, model structure, or state transitions, not merely when a model uses biological terminology or attention mechanisms.

## Foundation models and cell-state transitions

### General cell-state representation

Cell-state representation models map sparse, batch-dependent expression data to reusable latent spaces that should preserve biological state while limiting technical variation [60, 61]. scGPT, Geneformer, scFoundation, UCE, and TranscriptFormer use distinct pretraining schemes to learn transferable cell or gene representations, including cross-tissue and cross-species settings [15–19]. Because their tokenization and objectives differ, the shared foundation-model label does not imply equivalent biological content or transfer capability.

Cell2Sentence serializes expression as gene sentences to interface with language models, whereas xVERSE learns batch-invariant representations and probabilistic whole-transcriptome generation from single-cell and spatial data [31, 32]. These approaches improve representation or generation, but neither textual fluency nor generative quality establishes that latent variables correspond to regulatory mechanisms.

More generally, large-scale pretraining can support transfer without identifying causal biology. Statistical dependencies and attention weights may mix direct regulation, indirect co-expression, abundance, cell type, and batch effects, so mechanistic claims require intervention-based evidence rather than latent-space interpretability alone.

### Perturbation-response prediction

An interactive virtual cell requires perturbation-responsive state transitions in addition to representation. Response models should condition on initial state, intervention, dose, time, and biological context. State emphasizes cross-context perturbation prediction; SCALE learns conditional population transport; X-Pert unifies genetic, chemical, combinatorial, and dose-dependent perturbations; and Tahoe-x1 learns large-scale gene, cell, and compound representations from drug-perturbation data [20–23]. These models move training toward intervention-rich corpora, but matching an endpoint distribution does not identify the temporal path or mechanism that produced it.

X-Cell combines diffusion-based state generation with molecular and phenotypic priors, while UniPert-G2CP transfers phenotype knowledge from genetic to chemical screens [24, 45]. Such transfer remains conditional: knockout and drug treatment differ in dose, target multiplicity, metabolism, and engagement, so mechanism-specific and independent validation remain necessary.

### Cross-modal and unified multi-omics modeling

Virtual cells must integrate modalities without treating cross-modal agreement as causal equivalence. GigaTIME predicts multiprotein spatial patterns from H&E images, illustrating the need to evaluate not only image similarity but also cellular composition, neighborhoods, functional readouts, and cross-center stability [62]. EpiFoundation and EpiAgent model chromatin accessibility; CLM-X links RNA and ATAC; HoloCell jointly models epigenomic, transcriptomic, and proteomic states; and CellHermes frames heterogeneous omics tasks as question answering [5, 8, 36–38]. Their cross-modal claims remain dependent on paired-data quality and validation design.

Protein measurements add a functional layer that transcriptomics alone cannot supply. CAPTAIN learns paired RNA–surface-protein representations, whereas ProteinTalks uses perturbation proteomics and neural ODEs to model dynamic protein networks [9, 14]. Evaluation should therefore distinguish transcriptional consistency from prediction of protein abundance, interactions, and functional state.

### Boundaries of model scale and generalization

Increasing data or parameter scale does not by itself guarantee improved biological generalization. Single-cell data retain batch effects, sampling noise, tissue bias, and inconsistent labels, so larger corpora can replicate existing distributions without adding new mechanistic information [63]. Zero-shot representation evaluations and perturbation benchmarks have shown that complex models may underperform highly variable genes, principal components, unchanged predictions, additive models, or linear baselines in particular settings [25, 26]. These findings do not invalidate foundation models; they show that claimed gains depend on splits, preprocessing, baselines, metrics, and whether the task contains genuinely nonadditive or context-specific effects.

OOD evaluation should distinguish unseen cells or states, donors, cell types or tissues, platforms, perturbations, doses, combinations, species, time, space, and modalities. Random splits can preserve cell-line, perturbation, or batch information and therefore do not establish transfer to unfamiliar biology. DenAdel *et al*. [64] found no clear general scaling law with data size, while VCBench and SCMBench show that model rankings vary across tasks and datasets [65, 66]. Model size, pretraining volume, and the foundation-model label therefore cannot replace explicit distribution-shift evaluation.

For comparison, this review groups methods by primary contribution into mechanistic/structured, transcriptomic foundation, multi-omics, perturbation/dynamic, spatial/multicellular, proteomic, EM/ultrastructure, and system/intervention categories. Figure 3 visualizes this taxonomy, Table 2 provides the representative landscape, Table 1 gives the operational LCM/AIVC comparison, and Fig. 4 summarizes capability emphasis; none is a performance ranking.

**Figure 4:**
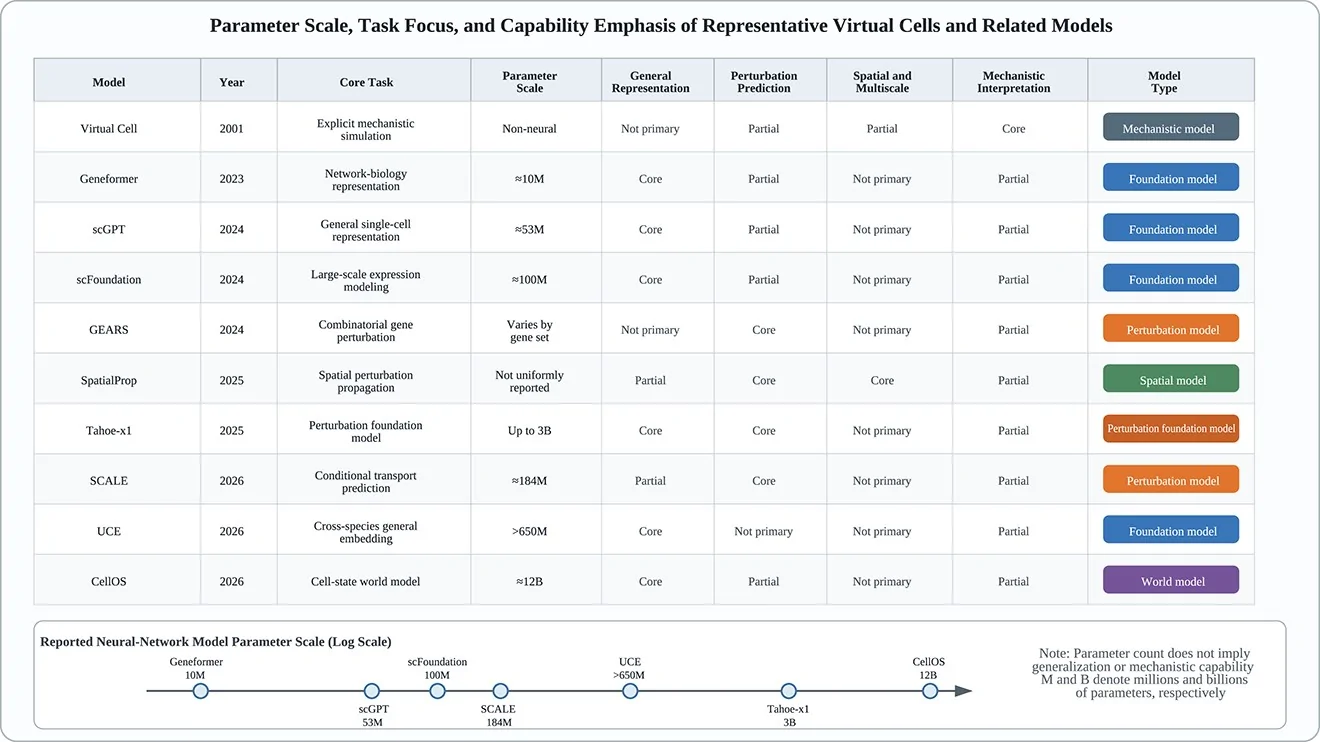
Parameter scale, task focus, and capability emphasis of representative virtual cells and related models. Core denotes a central stated objective or central implemented capability supported by direct demonstration in the original source. Partial denotes a demonstrated but secondary, conditional, limited-scope, or downstream-dependent capability. Not primary denotes a capability that is not a central objective of the reported model and does not mean that the model is incapable of it. The coding represents capability/research emphasis, not OOD maturity, evidence strength, independent validation, baseline superiority, or model ranking. Model-reference mapping: Virtual Cell [2]; Geneformer [16]; scGPT [15]; scFoundation [17]; GEARS [42]; SpatialProp [49]; Tahoe-x1 [23]; SCALE [20]; UCE [18]; CellOS [56]. Reported parameter counts are architecture-specific and are not directly comparable across models.

### Boundaries between large cellular models and AI virtual cells

Recent Reviews, Perspectives, Comments, and other conceptual articles use overlapping but nonidentical criteria for LCMs and AIVCs [1, 67–71]. Across these viewpoints, transferable representation is an important foundation, while stronger virtual-cell claims increasingly emphasize perturbation prediction, state transition, temporal dynamics, multimodal and spatial integration, mechanistic or causal interpretability, experimentally testable prediction, and experimental feedback. The relative weight placed on these properties differs across publications, so no single field-wide boundary should be inferred from terminology alone.

A representation model does not automatically constitute a complete virtual cell, and a perturbation predictor does not automatically constitute a complete virtual cell. Both can nevertheless provide important enabling or predictive components of virtual-cell development. For analytical comparison in this review, LCMs are treated as systems whose central contribution is transferable state representation, whereas AIVCs place additional emphasis on perturbation-responsive transitions, conditional or temporal context, and outputs that can be experimentally tested. This is an operational convention adopted in this review rather than an established field-wide taxonomy.

Table 1 structures this operational comparison using functional emphasis rather than mandatory architectural requirements. Figure 4 provides a representative capability comparison. Its coding describes research emphasis and direct demonstrations in the cited sources rather than a performance ranking or a test of whether a model is a “true” virtual cell.

## Virtual systems at spatial, multicellular, and developmental scales

### Dynamic interactions in cell populations

Tissue cells respond to ligands, contact, metabolic competition, and mechanics rather than fixed external conditions. CellTRIP models cell populations as interacting agents with reinforcement learning and self-attention for spatiotemporal prediction [51]. This can represent heterogeneous, context-dependent transitions, but learned actions and interaction weights remain statistical unless linked to measurable communication or biophysical variables and tested by intervention. Combining multi-agent learning with explicit signaling, metabolic, and mechanical constraints may therefore improve interpretability and validation.

### Perturbation propagation in tissue space

Spatial foundation models have evolved from simply incorporating coordinate information to explicitly modeling spatial context. Nicheformer is pretrained on both dissociated single-cell and spatial omics datasets, attempting to jointly encode cell identity and the local tissue environment [6]; SToFM decomposes tissue macrostructure, microenvironment, and gene expression into multiscale subslices for pretraining [10]; STARNet combines spatial RNA and ATAC information to infer region-specific gene regulatory networks via heterogeneous hypergraphs [48]. These three approaches represent three complementary directions: spatial representation, multiscale spatial pretraining, and spatial regulatory networks.

SpatialProp focuses on the propagation of perturbations at the tissue scale. This method represents spatially resolved single-cell transcriptomes as a cellular graph, predicts gene expression based on the local microenvironment, and propagates the effects of local perturbations to other cells within the tissue [49]. Its CausalInteractionBench utilizes cell–cell interactions with known directions to assess whether the model recovers directionally consistent communication relationships. This evaluation is closer to causal inference than a simple comparison of expression correlations, but it still does not constitute causal proof. Rigorous validation requires controlled perturbations, temporal sequence, spatial blocking, or independent tissue experiments.

SpaRx provides a complementary spatial intervention example. It uses graph-based domain adaptation to transfer pharmacogenomic drug-response knowledge to single-cell spatial transcriptomic data and predicts spatially heterogeneous drug sensitivity within tissue [50]. It was retained in the revised representative landscape because spatially resolved intervention-response prediction is a central technical contribution under the same revision-stage category criterion applied to other spatial and perturbation methods.

VirTues further organizes the spatial proteome into hierarchical representations such as proteins, cells, niches, and tissues, and supports cross-panel transfer, spatial biomarker discovery, and patient stratification [53]. This work provides a relatively clear example of a multiscale representation, but whether its cross-level state transitions are interpretable still requires verification through interventions at different scales and longitudinal data.

### Predictive virtual embryos

Predictive virtual embryos extend virtual-cell ideas to development by linking cell state, lineage, spatial position, movement, and tissue morphology. Cao *et al*. [52] describe this as a research vision rather than an implemented, systematically benchmarked model. A testable system would need to model division, migration, fate, morphogens, mechanics, and tissue boundaries with aligned timescales and uncertainty; transcriptional prediction alone cannot establish correct developmental structure.

### World models, cross-scale modeling, and system integration

CellOS uses joint-embedding prediction to learn multiple views of cellular state and is presented as a cellular world-model direction [56]. This may support unified representation, querying, and state prediction, but the current preprint still requires independent verification of scope and reproducibility.

Multimodal input alone is not equivalent to cross-scale modeling. A conceptual bidirectional hierarchy can be written as


(8) \begin{eqnarray*}& z^{mol} \rightarrow z^{cell} \rightarrow z^{pop} \rightarrow z^{tissue}\end{eqnarray*}

and


(9) \begin{eqnarray*}& z^{tissue} \rightarrow z^{pop} \rightarrow z^{cell} \rightarrow z^{mol}.\end{eqnarray*}

A cross-scale virtual system must specify interpretable interfaces through which molecular changes alter cellular behavior and tissue structure, and through which tissue context feeds back to lower levels. These mappings, not merely the presence of several modalities in one network, require joint experimental validation.

### Volume electron microscopy and subcellular ultrastructure

Volume EM contributes 3D organelle geometry, membrane contacts, and network topology that are not captured by transcriptomic state alone. Current EM foundation models remain enabling components rather than complete virtual cells: EM-DINO/OmniEM support restoration and segmentation, EMCFsys combines restoration, segmentation, and sparse 3D reconstruction, and DF5T unifies several restoration tasks [11–13]. An ultrastructure-aware virtual cell would need to align these structural features with molecular and metabolic states and allow them to participate in state transitions, rather than treating EM as static imagery.

### System-level modeling and intervention design

The Virtual Yeast Perspective organizes mechanistic knowledge, ultrastructure, and dynamic omics as data pillars for a future system-level model, but it does not report a complete implemented virtual cell [54]. GEMGen generates molecules for target cellular phenotypes, whereas SequenTx uses cellular-state models and reinforcement learning to design sequential drug interventions with *in vitro* and *in vivo* testing [46, 55]. These examples have different evidence profiles: candidate generation, model-guided intervention selection, independent experimental validation, and closed-loop updating are distinct claims and are evaluated separately in the later discussion of experimental validation and feedback.

## Data foundations, open resources, and reproducibility

### Data resources, prior knowledge, and coverage boundaries

Data coverage remains a major constraint. More cells improve statistical stability but cannot replace diversity in perturbations, contexts, dose, time, spatial location, and measurement platforms. For example, Stack uses scBaseCount together with CELLxGENE and Parse PBMC resources, while the Arc Virtual Cell Atlas integrates scBaseCount, Tahoe-100M, Virtual Cell Challenge data, and other resources [43, 72, 73].

These resources have different provenance roles. Primary measurements, standardized compilations, knowledge bases, derived benchmarks, model weights, and reproduction packages should not be counted as independent experimental data. Overlap among CELLxGENE, scBaseCount, Arc Atlas, Tahoe resources, and model-specific corpora can also inflate apparent training scale or external validation when the same parent studies or cells are reused.

Table 3 therefore separates experimental data, prior knowledge, and derived evaluation resources and adds concise provenance flags. Known overlap is reported only for traceable parent/derived relationships; potential overlap requires a source-level rationale; otherwise the relationship is recorded as not ascertainable. Supplementary Table S4 provides the corresponding provenance audit and implications for training/evaluation independence.

**Table 3:** Data, prior knowledge, provenance, and derived evaluation resources for virtual-cell modeling, reverified through 11 September 2026.

Data resource	Resource type	Modality and coverage	Primary use	Public access	Reference	Provenance/overlap note
**I. Experimental data and standardized atlases**						
**1. Observational atlases and general corpora**						
CELLxGENE Discover and Census	Standardized compilation	Single-cell transcriptomes from humans and model organisms; continuously updated	Data retrieval, unified access, pretraining corpus construction, and cross-dataset validation	https://cellxgene.cziscience.com	[74]	Parent/compilation source for many public single-cell datasets; overlap with downstream compilations must be checked at study/cell level.
scBaseCount	Standardized compilation	Current official release: >502 million cells across 27 organisms and 75 tissues; continuously expanding	Unified preprocessing, cross-species pretraining, and data lineage tracking	https://github.com/ArcInstitute/scBaseCount	[72]	Standardized compilation derived from public single-cell studies; some source studies may also appear in CELLxGENE-derived corpora.
Arc Virtual Cell Atlas	Integrated compilation	Integrated Atlas including current scBaseCount (>502 million cells), Tahoe-100M (100 million cells), and additional public/perturbation resources	Unified download, model training, and open evaluation	https://arcinstitute.org/tools/virtualcellatlas	[73]	Integrated compilation that includes scBaseCount, Tahoe-100M, and Virtual Cell Challenge resources; not independent of those components.
Tabula Sapiens	Primary atlas	Nearly 500 000 human cells from 24 tissues and organs	Cross-tissue cell-type reference and background transfer	https://tabula-sapiens.sf.czbiohub.org	[75]	Not ascertainable from the retained comparative record; see Supplementary Table S4 where relevant.
HuBMAP	Primary atlas and portal	Multi-organ single-cell, spatial multi-omics, and imaging; continuously updated	Tissue microenvironment, spatial structure, and 3D atlas validation	https://portal.hubmapconsortium.org	[76]	Not ascertainable from the retained comparative record; see Supplementary Table S4 where relevant.
TCGA	Primary cohorts and compilation	>11 000 tumor cases across 33 cancer types and multiple omics	Tumor context, patient stratification, and external clinical association	https://portal.gdc.cancer.gov	[77]	Not ascertainable from the retained comparative record; see Supplementary Table S4 where relevant.
CCLE	Cell-line atlas	Molecular omics and drug-response data for >1100 cancer cell lines	Cell-line context, drug response, and cross-platform validation	https://depmap.org/portal	[78]	Not ascertainable from the retained comparative record; see Supplementary Table S4 where relevant.
**2. Drug and environmental perturbations**						
Tahoe-100M	Primary screen and processed dataset	>100 million single-cell profiles; 50 cancer cell lines and about 1,100 small molecules	Drug response, dose response, and cross-cell-context prediction	https://huggingface.co/datasets/tahoebio/Tahoe-100M	[23]	Primary perturbation screen; also incorporated into Arc Virtual Cell Atlas and used by multiple model/benchmark resources.
Sci-Plex	Primary screen	About 650 000 cells, three cancer cell lines, and 188 compounds	Multidose chemical perturbation, cell state, and pathway response	https://github.com/coletrapnell-lab/sci-plex	[79]	Not ascertainable from the retained comparative record; see Supplementary Table S4 where relevant.
Parse 10M PBMCs	Primary screen	About 9.7 million cells from 12 donors under 90 cytokine stimulations	Immune context, donor differences, and cytokine response	https://www.parsebiosciences.com/datasets/10-million-human-pbmcs-in-a-single-experiment/	[80]	Primary screen; also used in model post-training/evaluation in some reported workflows.
LINCS L1000	Primary screen and compilation	About 1.3 million perturbation profiles with 978 directly measured transcripts	Compound and gene-perturbation search, pathway connectivity, and external validation	https://clue.io	[81]	Not ascertainable from the retained comparative record; see Supplementary Table S4 where relevant.
**3. Genetic and combinatorial perturbations**						
Virtual Cell Challenge 2025	Primary screen and benchmark	About 300 000 H1 cells covering 300 CRISPRi targets	Prediction of held-out perturbations and independent competition evaluation	https://virtualcellchallenge.org/datasets	[82, 83]	Primary screen and benchmark; incorporated into Arc Virtual Cell Atlas.
Replogle whole-genome Perturb-seq	Primary screen	>2.5 million human cells with genome-scale expression profiles	Gene function, regulatory networks, and state-transition learning	https://gwps.wi.mit.edu	[84]	Not ascertainable from the retained comparative record; see Supplementary Table S4 where relevant.
Adamson Perturb-seq	Primary screen	CRISPR screen of the unfolded-protein response; common processed version has about 68 600 cells and 86 perturbations	Single-gene effects, stress pathways, and perturbation-prediction benchmarks	https://ncbi.nlm.nih.gov/geo/query/acc.cgi?acc=GSE90546	[85]	Not ascertainable from the retained comparative record; see Supplementary Table S4 where relevant.
Dixit Perturb-seq	Primary screen	About 200 000 immune cells covering transcription factors and immune pathways	Pooled CRISPR screening and regulatory-network analysis	https://ncbi.nlm.nih.gov/geo/query/acc.cgi?acc=GSE90063	[86]	Not ascertainable from the retained comparative record; see Supplementary Table S4 where relevant.
Norman combinatorial perturbations	Primary screen	Common processed version has about 91 200 cells with single- and dual-gene perturbations	Genetic interactions, nonadditive effects, and combinatorial extrapolation	https://ncbi.nlm.nih.gov/geo/query/acc.cgi?acc=GSE133344	[87]	Not ascertainable from the retained comparative record; see Supplementary Table S4 where relevant.
Nadig dataset	Primary screen	Whole-transcriptome Perturb-seq in Jurkat and HepG2 cells, mainly covering common essential genes	Essential-gene effects across cell lines and perturbation generalization	https://ncbi.nlm.nih.gov/geo/query/acc.cgi?acc=GSE264667	[88]	Not ascertainable from the retained comparative record; see Supplementary Table S4 where relevant.
Jiang multicell-line screen	Primary screen	>1500 perturbations across six cell lines and five stimulus environments	Pathway signatures, environment-dependent effects, and cross-context prediction	https://ncbi.nlm.nih.gov/geo/query/acc.cgi?acc=GSE281048	[89]	Not ascertainable from the retained comparative record; see Supplementary Table S4 where relevant.
X-Atlas and Orion	Primary screen and processed dataset	Genome-wide Perturb-seq in HCT116 and HEK293T; public dataset about 521 GB	High-coverage genetic perturbation and cross-cell-line validation	https://doi.org/10.25452/figshare.plus.29190726	[90]	Not ascertainable from the retained comparative record; see Supplementary Table S4 where relevant.
scPerturb	Standardized compilation	Unified curation of 44 public single-cell perturbation studies across transcriptomics, proteomics, and epigenomics	Cross-study quality control and benchmarking	https://scperturb.org	[91]	Derived standardized compilation of public perturbation studies; source-study overlap should be expected when those studies are separately used.
**4. Proteins, imaging, and subcellular ultrastructure**						
Human Protein Atlas Cell Atlas	Primary measurements and atlas	Antibody imaging, transcriptomics, and mass-spectrometry proteomics; 12 003 proteins localized to 30 subcellular structures	Protein localization, subcellular state, and cross-modal validation	https://proteinatlas.org/about/download	[92]	Not ascertainable from the retained comparative record; see Supplementary Table S4 where relevant.
OpenCell	Primary measurements and atlas	Live-cell imaging and immunoprecipitation mass spectrometry for 1310 endogenously tagged proteins	Protein localization, complexes, and interaction networks	https://opencell.sf.czbiohub.org/download	[93]	Not ascertainable from the retained comparative record; see Supplementary Table S4 where relevant.
JUMP Cell Painting	Primary screen and feature dataset	About 3 million five-channel images and 75 million single-cell features	Alignment of morphological phenotypes with genetic and chemical perturbations	https://registry.opendata.aws/cellpainting-gallery	[94]	Not ascertainable from the retained comparative record; see Supplementary Table S4 where relevant.
OpenOrganelle	Primary measurements and segmentations	3D focused-ion-beam scanning EM with 4 and 8 nm voxels and multiple organelle types	Ultrastructural representation, 3D geometry, and organelle-relation modeling	https://openorganelle.janelia.org	[95]	Not ascertainable from the retained comparative record; see Supplementary Table S4 where relevant.
**5. Pharmacological phenotypes and external validation**						
GDSC	Pharmacological screen	1001 cancer cell lines and sensitivity measurements for hundreds of anticancer drugs	Drug-response modeling, pharmacogenomic association, and external validation	https://cancerrxgene.org	[96]	Not ascertainable from the retained comparative record; see Supplementary Table S4 where relevant.
PRISM	Pharmacological screen	Activity profiles for 4518 drugs across 578 human cancer cell lines	Large-scale drug repositioning and cross-cell-line validation	https://depmap.org/portal/prism	[97]	Not ascertainable from the retained comparative record; see Supplementary Table S4 where relevant.
DrugComb	Standardized compilation	Initial release included 437 932 drug-combination experiments; continuously updated	Drug synergy, antagonism, and combination-therapy validation	https://drugcomb.org	[98]	Not ascertainable from the retained comparative record; see Supplementary Table S4 where relevant.
Yeast G2T Atlas	Primary screen	3500 mutants under salt stress, covering 75% of nonessential genes	Genetic and environmental perturbation, state plasticity, and mechanistic validation	https://doi.org/10.1038/s41467-025-57600-4	[99]	Not ascertainable from the retained comparative record; see Supplementary Table S4 where relevant.
**II. Prior knowledge: functional annotation and mechanistic priors**						
UniProt	Manual curation and computational annotation	Protein sequence, function, localization, domains, and variants	Protein-function constraints, gene-to-protein mapping, and interpretation	https://uniprot.org	[100]	Not ascertainable from the retained comparative record; see Supplementary Table S4 where relevant.
Gene Ontology	Ontology and annotation	Molecular function, biological process, and cellular component	Functional modules, pathway enrichment, and mechanistic-consistency evaluation	https://geneontology.org	[101]	Not ascertainable from the retained comparative record; see Supplementary Table S4 where relevant.
Reactome	Pathway knowledge base	Curated human reactions and pathways with cross-species mapping	State-transition constraints, pathway-level interpretation, and mechanistic validation	https://reactome.org	[102]	Not ascertainable from the retained comparative record; see Supplementary Table S4 where relevant.
STRING	Interaction knowledge base	Multispecies protein-interaction networks integrating experiments, databases, and text mining	Protein-interaction priors, graph construction, and network validation	https://string-db.org	[103]	Not ascertainable from the retained comparative record; see Supplementary Table S4 where relevant.
CORUM	Complex knowledge base	7969 experimentally supported mammalian protein complexes (CORUM release 5.3)	Complex constraints, protein modules, and subcellular functional interpretation	https://mips.helmholtz-muenchen.de/corum	[104]	Not ascertainable from the retained comparative record; see Supplementary Table S4 where relevant.
HGNC and GENCODE	Gene-naming and annotation standards	Gene symbols, transcripts, and reference-gene annotation	Identifier unification, feature alignment, and version control	https://genenames.org; https://gencodegenes.org	[105, 106]	Not ascertainable from the retained comparative record; see Supplementary Table S4 where relevant.
Ensembl BioMart	Genome annotation and query	Multispecies genes, transcripts, orthology, and programmable filtering	Cross-species mapping, gene-set curation, and feature annotation	https://mart.ensembl.org	[107]	Not ascertainable from the retained comparative record; see Supplementary Table S4 where relevant.
Saccharomyces Genome Database	Model-organism knowledge base	Curated genome, function, phenotype, interaction, and pathway information	Mechanistic priors, parameter sources, and validation for system-level models	https://yeastgenome.org/downloads	[108]	Not ascertainable from the retained comparative record; see Supplementary Table S4 where relevant.
				**III. Derived evaluation resources**		
GEARS common processed collection	Derived benchmark	Standard task versions derived from Adamson, Norman, Replogle, and other public screens	Evaluation of single-gene, combinatorial, and unseen perturbations	https://github.com/snapstanford/GEARS	[42, 84, 85, 87]	Derived from Adamson, Norman, Replogle, and other public perturbation screens; not an independent experimental source relative to those parents.
GeneTAK	Derived benchmark	Training and task collections derived from Tahoe-100M and related resources	Drug-response prediction and knowledge-enhanced retrieval	https://github.com/GENTEL-lab/VCWorld	[109]	Derived benchmark using Tahoe-100M and related resources; independence depends on the compared model’s pretraining/evaluation corpus.
VCBench	Derived benchmark	Seven datasets from six studies, comparing 11 methods	Evaluation of unseen cells, unseen perturbations, and multi-dataset robustness	https://github.com/maoxx241/VCBench	[66]	Derived benchmark constructed from previously published perturbation datasets; parent-study overlap must be checked for each evaluated model.
MVCBench	Derived multimodal benchmark	Drug-induced transcriptomic and morphological phenotypes; multimodal ID/OOD evaluation	Benchmarking representation and prediction methods under multimodal and OOD settings	https://github.com/QSong-github/MVCBench	[110]	Derived benchmark; benchmark data and parent-study relationships should be checked against each model’s training corpus before calling an evaluation independent.

Note. Resources were scientifically reverified through 11 September 2026 and may continue to change. Derived benchmarks are not counted again as independent experimental data. Abbreviations: CCLE, Cancer Cell Line Encyclopedia; CORUM, Comprehensive Resource of Mammalian Protein Complexes; CRISPR, clustered regularly interspaced short palindromic repeats; CRISPRi, CRISPR interference; EM, electron microscopy; G2T, genotype-to-transcriptome; GB, gigabyte; GDSC, Genomics of Drug Sensitivity in Cancer; HGNC, HUGO Gene Nomenclature Committee; HuBMAP, Human BioMolecular Atlas Program; JUMP, Joint Undertaking in Morphological Profiling; LINCS, Library of Integrated Network-based Cellular Signatures; nm, nanometer; PBMC, peripheral blood mononuclear cell; PRISM, Profiling Relative Inhibition Simultaneously in Mixtures; STRING, Search Tool for the Retrieval of Interacting Genes/Proteins; TCGA, The Cancer Genome Atlas.

### Model openness and licensing

Reproducibility must be assessed separately for papers, code, weights, licenses, data versions, vocabularies, preprocessing, checkpoints, and inference settings. Code release does not imply released weights or unrestricted use, and a project page is not equivalent to a complete reproduction package.

Because repositories change rapidly, revision-stage mutable-resource verification was performed on 3 September 2026, and current publication and resource status was rechecked on 11 September 2026. Release/tag, revision, model-version, and licensing details are recorded where ascertainable in Supplementary Table S3. Statements such as “no official author release identified as of 11 September 2026” are deliberately time-bounded and do not establish that a resource does not exist. Table 4 summarizes the availability of code and model weights and the licensing status of representative virtual-cell models.

**Table 4:** Open resources and licensing status of models cited in this review, reverified on 11 September 2026.

Model	Reference	GitHub URL	Hugging Face URL	License and terms of use	Verification date
**Mechanistic models and structured prediction**					
Virtual Cell	[2]	https://github.com/virtualcell/vcell	Not applicable	Code: MIT; weights: not applicable	11 September 2026
Whole-cell model	[4]	https://github.com/CovertLab/WholeCell	Not applicable	Code: MIT; weights: not applicable	11 September 2026
PhysiCell	[3]	https://github.com/MathCancer/PhysiCell	Not applicable	Code: BSD-3-Clause; weights: not applicable	11 September 2026
SCALE	[20]	No official author release identified as of 11 September 2026	No official author release identified as of 11 September 2026	No official author release identified as of 11 September 2026	11 September 2026
RegFormer	[7]	https://github.com/BGIResearch/RegFormer	No separate official Hugging Face page identified as of 11 September 2026	MIT	11 September 2026
				**Transcriptomic foundation models**	
scBERT	[27]	https://github.com/TencentAILabHealthcare/scBERT	No official author release identified as of 11 September 2026	GPL-3.0	11 September 2026
Geneformer	[16]	No separate repository	https://huggingface.co/ctheodoris/Geneformer	Apache-2.0	11 September 2026
CellPLM	[28]	https://github.com/OmicsML/CellPLM	No official Hugging Face page identified as of 11 September 2026	BSD-2-Clause; repository provides pretrained checkpoint links	11 September 2026
scGPT	[15]	https://github.com/bowang-lab/scGPT	https://huggingface.co/wanglab/scGPT-human	Code: MIT; separate weight license not stated	11 September 2026
scFoundation	[17]	https://github.com/biomap-research/scFoundation	https://huggingface.co/genbio-ai/scFoundation	Code: Apache-2.0; original weights restricted to noncommercial research; Hugging Face distribution provides the encoder only	11 September 2026
GeneCompass	[29]	https://github.com/xCompass-AI/GeneCompass	No official author release identified as of 11 September 2026	Repository has no stated license	11 September 2026
scMulan	[30]	https://github.com/SuperBianC/scMulan	No official author release identified as of 11 September 2026	MIT	11 September 2026
Cell2Sentence	[31]	https://github.com/vandijklab/cell2sentence	No unified model page found	Apache-2.0	11 September 2026
xVERSE	[32]	https://github.com/jichunxie/xVERSE_code	No official author release identified as of 11 September 2026	GPL-3.0	11 September 2026
UCE	[18]	https://github.com/snapstanford/UCE	No official author release identified as of 11 September 2026	MIT	11 September 2026
TranscriptFormer	[19]	https://github.com/czi-ai/transcriptformer	No separate official Hugging Face page identified as of 11 September 2026	MIT	11 September 2026
				**Epigenomics and unified multi-omics**	
scMVP	[33]	https://github.com/bm2-lab/scMVP	No official Hugging Face page identified as of 11 September 2026	License status not clearly stated in the available repository metadata	11 September 2026
scCLIP	[34]	https://github.com/jsxlei/scCLIP	No official author release identified as of 11 September 2026	Repository has no stated license	11 September 2026
scmFormer	[35]	https://github.com/zhanglabwbgcas/scmFormer	No official author release identified as of 11 September 2026	Repository has no stated license	11 September 2026
EpiFoundation	[36]	https://github.com/UCSC-VLAA/EpiFoundation	No official Hugging Face page identified as of 11 September 2026	License status not clearly stated in the available repository metadata	11 September 2026
EpiAgent	[5]	https://github.com/xy-chen16/EpiAgent	No official Hugging Face page identified as of 11 September 2026	MIT; v1.0.0 official release provides source code, pretrained model, and example datasets	11 September 2026
CellHermes	[37]	https://github.com/theislab/CellHermes	No official author release identified as of 11 September 2026	Repository has no stated license	11 September 2026
CAPTAIN	[14]	https://github.com/iamjiboya/CAPTAIN	No official author release identified as of 11 September 2026	Repository has no stated license	11 September 2026
CLM-X	[38]	https://github.com/HIM-AIM/CLM-X	No official Hugging Face page identified; checkpoints are linked from the official repository	MIT; official repository links training data/checkpoints via Zenodo	11 September 2026
HoloCell	[8]	https://github.com/bjzgcai/HoloCell	No official Hugging Face page identified as of 11 September 2026	Repository currently exposes a GPL-3.0 license file; README notes release terms for source/weights should be checked before reuse	11 September 2026
SCARF	[39]	https://github.com/cbmigroup/scarf	No official author release identified as of 11 September 2026	Code: GPL-3.0; weight license not stated	11 September 2026
				**Perturbation and dynamic models**	
CPA	[40]	https://github.com/theislab/CPA	No official Hugging Face page identified as of 11 September 2026	BSD-3-Clause	11 September 2026
Neural OT	[41]	https://github.com/bunnech/cellot	No official author release identified as of 11 September 2026	Repository has no stated license	11 September 2026
GEARS	[42]	https://github.com/snap-stanford/GEARS	No official Hugging Face page identified as of 11 September 2026	MIT	11 September 2026
State	[21]	https://github.com/ArcInstitute/state	https://huggingface.co/collections/arcinstitute/state	Code: CC BY-NC-SA 4.0; model weights subject to a separate noncommercial license	11 September 2026
Stack	[43]	https://github.com/ArcInstitute/stack	https://huggingface.co/collections/arcinstitute/stack	Code: CC BY-NC-SA 4.0; model weights subject to separate noncommercial terms	11 September 2026
X-Pert	[22]	https://github.com/Chen-Li-17/X-Pert	No official Hugging Face page identified as of 11 September 2026	MIT	11 September 2026
Tahoe-x1	[23]	https://github.com/tahoebio/tahoe-x1	https://huggingface.co/tahoebio/Tahoe-x1	Code and weights: Apache-2.0	11 September 2026
AlphaCell	[44]	No official author release identified as of 11 September 2026	No official author release identified as of 11 September 2026	No official author release identified as of 11 September 2026	11 September 2026
X-Cell	[24]	https://github.com/Xaira-Therapeutics/X-Cell	https://huggingface.co/Xaira-Therapeutics/X-Cell	CC BY-NC-SA 4.0	11 September 2026
UniPert-G2CP	[45]	https://github.com/TencentAILabHealthcare/UniPert	No official Hugging Face page identified as of 11 September 2026	GPL-3.0; v1.0.0 release available	11 September 2026
SequenTx	[46]	https://github.com/DELTA-TJ/SequenTx	No official Hugging Face page identified as of 11 September 2026	MIT	11 September 2026
				**Spatial, multicellular, and proteomic models**	
NicheCompass	[47]	https://github.com/Lotfollahi-lab/nichecompass	No official Hugging Face page identified as of 11 September 2026	BSD-3-Clause; tagged releases available	11 September 2026
SToFM	[10]	https://github.com/PharMolix/SToFM	No official author release identified as of 11 September 2026	Repository has no stated license	11 September 2026
Nicheformer	[6]	https://github.com/theislab/nicheformer	https://huggingface.co/theislab/Nicheformer	BSD-3-Clause	11 September 2026
STARNet	[48]	https://github.com/DBinary/STARNet	No official author release identified as of 11 September 2026	Repository has no stated license	11 September 2026
SpatialProp	[49]	https://github.com/abuendia/spatial-prop	No official Hugging Face page identified as of 11 September 2026	MIT	11 September 2026
CellTRIP	[51]	https://github.com/daifengwanglab/CellTRIP	No official author release identified as of 11 September 2026	Repository has no stated license	11 September 2026
Predictive virtual embryos	[52]	Not applicable	Not applicable	Conceptual framework; model licensing is not applicable	11 September 2026
VirTues	[53]	https://github.com/bunnelab/virtues	Official model weights are linked from the project repository; individual weight repositories have model-specific terms	Code: MIT; some released weights use additional model-specific terms	11 September 2026
ProteinTalks	[9]	https://github.com/guomics-lab/PTV-1/tree/main/ProteinTalks	No official Hugging Face page identified as of 11 September 2026	No explicit model-weight license identified from the retained public repository record	11 September 2026
				**Electron-microscopy models and system-level frameworks**	
EM-DINO and OmniEM	[11]	No official author release identified as of 11 September 2026	No official author release identified as of 11 September 2026	No official author release identified as of 11 September 2026	11 September 2026
EMCFsys	[12]	No official author release identified as of 11 September 2026	No official author release identified as of 11 September 2026	No official author release identified as of 11 September 2026	11 September 2026
DF5T	[13]	https://github.com/dumengze/DF5T	No official Hugging Face page identified as of 11 September 2026	Fine-tuned models are linked from the official repository; license not clearly stated in the available repository metadata	11 September 2026
Virtual yeast	[54]	Not applicable	Not applicable	Conceptual framework; model licensing is not applicable	11 September 2026
GEMGen	[55]	https://github.com/DLS5-Omics/GEMGen	No official Hugging Face page identified; pretrained checkpoints are linked from the official repository	Code repository reports Apache-2.0; pretrained checkpoints are distributed separately	11 September 2026
CellOS	[56]	No official author release identified as of 11 September 2026	No official author release identified as of 11 September 2026	No official author release identified as of 11 September 2026	11 September 2026

Note. Revision-stage mutable-resource verification was performed on 3 September 2026, and current mutable resource status was rechecked on 11 September 2026. ”No official author release identified as of 11 September 2026” is a time-bounded search result, not evidence that a resource does not exist. Release/tag/revision details are recorded in Supplementary Table S3. Abbreviations: BSD, Berkeley Software Distribution; CC BY-NC-SA, Creative Commons Attribution-NonCommercial-ShareAlike; GPL, GNU General Public License; MIT, Massachusetts Institute of Technology; URL, uniform resource locator.

### Parameter scale, training data, and compute disclosure

Table 5 separates architecture from primary training objectives while summarizing parameter scale, training data, hardware, and duration. These quantities are reported unevenly and are architecture-specific; training-cell counts may also refer to raw profiles, unique cells, processed examples, or repeated sampling. Parameter count is therefore neither a reproducibility metric nor a substitute for performance, OOD generalization, or experimental validation.

**Table 5:** Architecture, primary training objectives, parameter scale, training data, and compute disclosure for models discussed in this review.

Model	Main architecture	Primary training objective(s)	Parameter scale	Training data	Hardware configuration and training duration
**Mechanistic models and structured prediction**					
Virtual Cell	ODEs, PDEs, stochastic reactions, particle-based and rule-based models	Not applicable	Not applicable	Determined by the specific biological model	No standardized training process
Whole-cell model	Modules for multiple biological processes with constraint-based simulation	Not applicable	Not applicable	Whole-cell knowledge base curated from the literature	No data pretraining
PhysiCell	Off-lattice multicellular agents with microenvironmental diffusion	Not applicable	Not applicable	User-defined cell and tissue parameters	No data pretraining
SCALE	Set-aware encoding, conditional latent transport, and endpoint prediction	Conditional flow matching/endpoint distribution transport for perturbation-response prediction	*∼* 184M	PBMC, Tahoe-100M, and Replogle-Nadig datasets	Hardware not reported; efficiency experiment: 243 s per epoch
RegFormer	Regulatory-network hierarchy and state-space model	GRN-guided representation pretraining with downstream cell-state and perturbation objectives; exact composite loss details follow the original source	Not reported	Single-cell transcriptomics and regulatory priors	Not reported
**Transcriptomic foundation models**					
scBERT	BERT-style encoding and expression-value binning	Masked-expression/BERT-style representation learning	Not reported	*∼* 1.1 million cells	Not reported
Geneformer	Expression-ranked sequence encoding and masked-gene prediction	Masked-gene prediction on expression-ranked gene sequences	*∼* 10M	*∼* 30 million cells	12 V100 32 GB GPUs, about 3 days
CellPLM	Cell-level Transformer with a Gaussian-mixture prior	Self-supervised cell representation pretraining with a Gaussian-mixture prior; downstream task adaptation	Not reported	Single-cell and spatial transcriptomics	Not reported
scGPT	Generative Transformer with joint gene and expression-value embeddings	Generative masked gene-expression/value prediction with task-specific adaptation	*∼* 53M	*∼* 33 million cells	Example: 10.3 million cells, 4 A100 GPUs, about 3 days for 6 epochs
scFoundation	Asymmetric masked autoencoder with Performer decoder	Masked expression reconstruction using an asymmetric autoencoding objective	*∼* 100M	>50 million cells	Not reported
GeneCompass	Cross-species BERT integrating regulatory knowledge	Masked gene prediction with cross-species regulatory-prior integration	*∼* 100M	*∼* 102 million human and mouse cells	32 NVIDIA A800 GPUs (4 *×* 8), about 9 days
scMulan	Prompt-driven generative Transformer	Autoregressive/prompt-conditioned generative modeling of cell sentences and task outputs	*∼* 368M	*∼* 10 million cells with metadata	Not reported
Cell2Sentence	Gene-sequence text conversion with adaptation of general language models	Autoregressive language modeling of serialized gene-expression sentences	Depends on base model	Single-cell corpora and task instructions	Not uniformly reported
xVERSE	Batch-invariant representation and probabilistic generation	Batch-invariant representation learning and probabilistic whole-transcriptome generation	Not reported	Single-cell and spatial transcriptomics	Not reported
UCE	Protein embeddings and chromosome-position-guided 33-layer Transformer	Not clearly reported in the available source	Over 650M	>36 million cells from over 300 datasets	24 A100 80 GB GPUs, 40 days
TranscriptFormer	Cross-species generative Transformer	Generative likelihood modeling of gene-expression profiles across species	*∼* 368M–542M trainable	57–112 million cells	Not reported
				**Epigenomics and unified multi-omics**	
scMVP	Multiview variational autoencoder with self-attention	Variational reconstruction with latent regularization for paired RNA–ATAC modeling	Not uniformly reported	Paired RNA and ATAC data	RTX 3090 testing reported; total duration not reported
scCLIP	Dual Transformer encoders with cross-modal contrastive learning	Cross-modal contrastive alignment of RNA and ATAC representations	Not uniformly reported	Paired RNA and ATAC data	Not reported
scmFormer	Multitask Transformer and cross-modal generation	Multitask masked/representation learning with cross-modal reconstruction or generation	Not uniformly reported	RNA, protein, and multi-omics data	Not reported
EpiFoundation	Peak-to-gene-aligned Transformer	Peak-to-gene-aligned representation learning with RNA-supervised objectives	Not reported	scATAC-seq with RNA supervision	Not reported
EpiAgent	Chromatin-accessibility sequence encoding with bidirectional attention	Self-supervised chromatin-accessibility representation learning with downstream perturbation/virtual-knockout adaptation	Not reported	Human scATAC corpus	Not reported
CellHermes	Low-rank adaptation of large language models for omics question answering	Task-conditioned language-model adaptation for omics question answering	Depends on base model	Transcriptomes, protein interactions, and task-specific text	Not reported
CAPTAIN	Joint Transformer for RNA and surface proteins	Joint RNA–protein representation learning with cross-modal protein inference objectives	Not reported	>4 million paired cells	FlashAttention-capable GPUs recommended; total runtime not reported
CLM-X	Multiway Transformer and cross-modal translation	Multimodal pretraining plus cross-modal translation and perturbation fine-tuning objectives	Not reported	RNA and ATAC data	Not reported
HoloCell	Three-omics generative foundation model for joint multimodal learning	Multimodal representation learning with iterative diffusion/remasking for cross-modal generation	Over 860M	*∼* 468 million omics profiles	Not reported
SCARF	Mamba with self-supervised and contrastive learning	Self-supervised representation learning with contrastive alignment	Not reported	X-Omics: about 2.7 million RNA and ATAC cells	Not reported
				**Perturbation and dynamic models**	
CPA	Disentangled autoencoder and dose–response modeling	Reconstruction plus disentanglement/adversarial regularization and dose-aware perturbation prediction	Not uniformly reported	Multiple drug and genetic-perturbation datasets	Not uniformly reported
Neural OT	Neural optimal transport and population-distribution mapping	Neural optimal-transport objective for mapping control to perturbed population distributions	Not uniformly reported	Paired or unpaired perturbation data	Not uniformly reported
GEARS	Perturbation embeddings, prior graphs, and graph neural networks	Supervised perturbation-response prediction with graph-informed representations	Depends on gene-set configuration	Perturb-seq datasets	Example: one GPU, 20 epochs
State	State encoder and Llama-based state-transition module	Supervised population/state-transition prediction across cellular contexts	Not reported	Nearly 170 million observational cells and >100 million perturbed cells	Not reported
Stack	Dual-axis cell-gene attention, gene-module tokens, and encoder-decoder architecture	Masked conditional cell-set prediction/in-context cross-condition reconstruction	76.7M–629M across reported variants; Stack-Large 217M	scBaseCount: about 149 million cells; CELLxGENE and Parse PBMC: about 55 million	Repository testing: H100 80 GB; training cluster and duration not reported
X-Pert	Dual-branch Transformer with perturbation-aware and cell encoders	Supervised perturbation-response prediction across genetic, chemical, combination, and dose conditions	Not reported	Genetic, chemical, and combinatorial perturbations	Not reported
Tahoe-x1	Perturbation Transformer with distributed FSDP	Perturbation-conditioned generative expression modeling/masked-expression prediction	70M, 1.3B, and 3B	*∼* 266 million cell profiles	Code requires A100 or newer GPU; actual cluster and runtime not reported
AlphaCell	Conditional flow matching and continuous state transition	Conditional flow matching for continuous perturbation-induced state transition	Not reported	Large-scale perturbation transcriptomics	Not reported
X-Cell	Set-level diffusion Transformer with cross-attention priors	Diffusion/denoising objective for perturbation-conditioned cell-state generation	55M to 4.9B	*∼* 25.6 million perturbed cells	Not reported
UniPert-G2CP	Unified representations and transfer learning	Cross-domain transfer/alignment from genetic perturbation phenotypes to chemical perturbation prediction	Not reported	Genetic and chemical screening data	Not reported
SequenTx	Reinforcement learning for sequential drug decisions	Reinforcement-learning objective for sequential treatment decisions coupled to a learned cellular-state model	Not uniformly reported	Tumor transcriptomics and drug-response data	Not uniformly reported
				**Spatial, multicellular, and proteomic models**	
NicheCompass	Graph autoencoder with communication priors	Graph-autoencoding/reconstruction with communication-program priors	Not uniformly reported	Spatial multi-omics	Not uniformly reported
SToFM	Three-level Transformer across macrostructure, microenvironment, and genes	Multiscale spatial representation pretraining across tissue, neighborhood, and gene levels	Not reported	SToCorpus-88M	Not reported
Nicheformer	Transformer for single-cell and spatial omics	Cellular reconstruction pretraining on dissociated and spatial omics	49.3M	SpatialCorpus-110M: >57M dissociated + 53M spatial cells across 73 tissues	3 compute nodes *×* 4 NVIDIA A100 40 GB GPUs (12 GPUs total), *∼*10 days
STARNet	Heterogeneous hypergraph for spatial RNA and ATAC	Spatial RNA–ATAC regulatory-network inference objective	Not uniformly reported	Spatial multi-omics	Not reported
SpatialProp	Spatial cell-graph neural network with error calibration	Supervised spatial perturbation-response prediction with error calibration	Not uniformly reported	Dataset-dependent spatial transcriptomics	Not reported
CellTRIP	Multi-agent reinforcement learning with self-attention	Multi-agent reinforcement-learning objective for spatiotemporal cell-state prediction	Not uniformly reported	Spatiotemporal single-cell states	Not reported
Predictive virtual embryos	Developmental-system modeling framework	Not applicable	Not applicable	Conceptual research program	Not applicable
VirTues	Marker-aware hierarchical spatial proteomics model	Marker-aware hierarchical representation learning with masked/reconstruction-based spatial-proteomic objectives	Not reported	Multiplatform spatial proteomics	Not reported
ProteinTalks	Neural ODE and dynamic protein networks	Neural-ODE trajectory fitting/perturbation-proteomics state-transition modeling	Not uniformly reported	Perturbation proteomics	Not reported
				**Electron-microscopy models and system-level frameworks**	
EM-DINO and OmniEM	Self-supervised visual encoding and unified EM pretraining	Self-supervised visual representation learning for EM restoration/segmentation transfer	Not reported	2D and 3D electron-microscopy corpora	Not reported
EMCFsys	Multitask EM foundation model with reconstruction decoder	Multitask EM restoration/reconstruction and segmentation objectives	Not reported	Multimodal and volume electron microscopy	Not reported
DF5T	Foundation model for EM image restoration	Image restoration objective spanning denoising, deblurring, super-resolution, inpainting, and isotropic reconstruction	Not reported	Volume EM and organelle images	Not reported
Virtual yeast	Modular whole-system engineering framework	Not applicable	Not applicable	Mechanistic knowledge, ultrastructure, and dynamic omics	Not applicable
GEMGen	Phenotype-conditioned small-molecule generator	Phenotype-conditioned molecular generation with compatibility/scoring objectives	Depends on base model	Perturbation transcriptomics and molecular corpora	Not reported
CellOS	Autoregressive language modeling, MoE extension, and latent alignment	Autoregressive expression modeling and joint-embedding predictive/latent-alignment objectives	*∼* 12B	*∼* 390.5 million single-cell transcriptomic profiles	Not reported

Note. Parameter counts and training configurations are architecture-specific. Not applicable covers mechanistic models or conceptual frameworks without unified training parameters. FSDP denotes fully sharded data parallelism; MoE denotes mixture of experts. Abbreviations: ATAC, assay for transposase-accessible chromatin; B, billion; BERT, bidirectional encoder representations from transformers; EM, electron microscopy; FSDP, fully sharded data parallelism; GB, gigabyte; GPU, graphics processing unit; M, million; MoE, mixture of experts; OT, optimal transport; PBMC, peripheral blood mononuclear cell; RNA, ribonucleic acid; scATAC, single-cell assay for transposase-accessible chromatin; scATAC-seq, single-cell assay for transposase-accessible chromatin sequencing.

Training objectives further delimit what a model is optimized to learn. Representation models use masked prediction, reconstruction, or autoregressive prediction; multimodal systems use contrastive alignment, cross-modal reconstruction, or generation; perturbation/dynamics models use supervised state prediction, optimal transport, flow matching, or diffusion-related objectives. These objectives cannot be converted directly into capability claims: reconstruction does not establish perturbation causality, masked prediction does not imply temporal dynamics, and in-distribution performance does not establish OOD generalization. Model-level objectives are reported in Table 5 without inferring unavailable losses.

## Evaluation, causal validation, and closed-loop experiments

### Multilevel evaluation framework

Virtual-cell evaluation requires multiple levels. Numerical reconstruction metrics, including mean squared error, correlation, negative log-likelihood, and distribution distance, measure agreement but not recovery of key biological processes. Biological-effect metrics include differentially expressed gene overlap, perturbation-effect correlation, pathway enrichment, transcription-factor activity, and cell composition. PDCorr and DE Overlap assess perturbation effects [20], but remain sensitive to thresholds, sample size, and statistical procedures.

External and OOD evaluation asks whether performance transfers across a genuine distribution shift such as individuals, cell types, tissues, perturbations, platforms, species, time, or spatial contexts. Independent experimental validation is different: a prediction is tested using a newly generated experiment rather than only a held-out computational test. Closed-loop experimental feedback is narrower still and requires model predictions to influence experiment selection, followed by use of the experimental result for model update, recalibration, or a subsequent decision.

To avoid implying a single maturity ladder, the revised review assesses five complementary, non-ordinal evidence dimensions. These dimensions are used as a practical analytical framework to separate five distinct questions that are often conflated in virtual-cell evaluation: whether a model or claim is sufficiently documented to be traced and examined; whether empirical performance has been demonstrated for the stated task; whether that performance transfers beyond the original evaluated distribution; whether mechanistic or causal claims receive direct support beyond predictive association; and whether model predictions have been tested in independently generated experiments. These questions correspond, respectively, to citable and retrievable documentation (DOC), task-specific empirical evidence (TASK), external and OOD generalization evidence (GEN), mechanistic and causal evidence (MECH), and independent experimental validation (EXP). The dimensions are not successive validation levels: TASK does not imply GEN; GEN does not imply MECH or EXP; strong MECH evidence does not require completion of every OOD test; and EXP does not imply closed-loop updating. Closed-loop experimental feedback remains reported separately. Table 6 defines the dimensions and their limitations, while Supplementary Table S2 applies the same evidence coding across the representative landscape. Publication form, model scale, and the technical taxonomy are reported separately from these dimensions.

**Table 6:** Evidence dimensions for virtual-cell assessment.

Evidence dimension	Definition	Indicative evidence	Supported conclusion	Important limitation
Citable and retrievable documentation (DOC)	A citable and retrievable conceptual or technical description sufficient for the reported model, system, framework, method, or resource to be identified and basically examined.	Original article/preprint/conference paper, technical report, versioned documentation, or equivalent source containing enough technical detail for identification and basic examination.	The reported object and its stated technical/conceptual basis can be examined.	DOC does not establish predictive validity, OOD generalization, causal validity, or experimental effectiveness; publication existence alone is insufficient.
Task-specific empirical evidence (TASK)	Direct empirical evaluation on the model’s stated task or evaluation conditions.	Defined task, data/splits, relevant baselines, metrics, repeated or otherwise interpretable evaluation.	The reported method shows empirical performance for the specified task/conditions.	TASK does not establish transfer beyond the evaluated distribution.
External and OOD generalization evidence (GEN)	Evidence under a genuine distribution shift or external setting relevant to the claim.	Held-out biological contexts such as donors, cell types, tissues, perturbations, doses/combinations, platforms, species, time, space, or cross-modal assays, with leakage checks where applicable.	Transfer/generalization is supported within the stated shifted setting.	GEN does not establish mechanistic/causal validity or independent experimental validation.
Mechanistic and causal evidence (MECH)	Mechanistic consistency, intervention-based structure, or causal-related evidence beyond predictive association.	Mechanistic equations/constraints, controlled interventions, dose-time structure, mediators, directional tests, competing-hypothesis evaluation, or other causal-related evidence.	Specified mechanistic or causal claims receive direct support.	MECH does not imply external generalization or independent experimental validation.
Independent experimental validation (EXP)	Model predictions are tested in independently generated experiments rather than only on same-dataset computational holdouts.	New CRISPR/drug experiments, organoids, live-cell or spatial experiments, animal studies, or other independently generated tests matched to the prediction.	The tested prediction has independent experimental support in the reported setting.	EXP does not automatically imply closed-loop model updating or broad OOD generalization.

Note. The dimensions are complementary rather than ordinal and should not be interpreted as a single cumulative scale. Closed-loop experimental feedback is reported separately as a system-level model–experiment interaction property; independent experimental validation does not imply closed-loop updating. Evidence coding used in Supplementary Table S2: Demonstrated; Limited; Not evaluated; Not reported; Not ascertainable; Not applicable; Not demonstrated.

### Design of out-of-distribution evaluation

Perturbation prediction benchmarks should clearly distinguish between different OOD tasks. Random cell splitting primarily tests in-distribution performance under closely matched conditions; unseen cell types test cross-context transfer; unseen perturbations test the extrapolation of perturbation representations; and unseen combined perturbations test whether the model can recognize nonadditive interactions; cross-laboratory and cross-platform testing, meanwhile, assess robustness to variations in batches and measurement mechanisms.

To separate additive from nonadditive effects, let $\Delta x_{A}$, $\Delta x_{B}$, and $\Delta x_{A + B}$ denote the observed effects of perturbations *A*, *B*, and their combination:


(10) \begin{eqnarray*}& \Delta x_{A + B} = \Delta x_{A} + \Delta x_{B} + \epsilon_{A,B}.\end{eqnarray*}

Here, $\epsilon _{A,B}$ is the nonadditive interaction. A metric dominated by $\Delta x_{A} + \Delta x_{B}$ may reward a simple additive model; interaction prediction should therefore be judged primarily by recovery of $\epsilon _{A,B}$.

A rigorous benchmark should also prevent information leakage. If different doses of the same drug, different compounds targeting the same target, or highly similar genes in the same pathway appear separately in the training and test sets, the test perturbations may still closely resemble the training data. A more rigorous evaluation should group data by target, pathway, chemical scaffold, cell lineage, and experimental batch, and report any potential overlap between the pretraining corpus and the test set.

The term zero-shot is not used consistently across studies, so Table 7 reports the biological axis of generalization rather than a single OOD label. The comparison distinguishes unseen cell states, donors, cell types or tissues, perturbations, doses and combinations, platforms or batches, species, temporal or spatial extrapolation, and cross-modal or cross-assay transfer where reported. It also records the evaluation setting or holdout, the main reported baseline, mechanistic or causal evidence, independent experimental validation, and uncertainty reporting. Simple baselines remain important because a complex model should establish biological gain beyond context or perturbation means and other low-complexity predictors. A same-dataset held-out test is not treated as independent experimental validation, and claims of external evaluation must be interpreted together with pretraining/evaluation provenance. Uncertainty, calibration, OOD detection, and abstention remain unevenly reported across the field.

**Table 7:** Generalization and validation evidence for major predictive virtual-cell and related models.

Model	Generalization type(s)	Evaluation setting/holdout/reported outcome	Main baseline	Mechanistic/causal evidence	Independent experimental validation	Uncertainty	Reference
SCALE	held-out cellular context; unseen/held-out perturbation	Evaluated on PBMC, Tahoe-100M, and Replogle-derived settings. Tahoe-100M includes held-out cell-line contexts with no data from those cell lines used during model development; Replogle-derived cross-context tests include held-out-context transfer with partial perturbation exposure where specified.	Simple predictive baselines reported in the source/benchmark comparisons	Limited: biology-guided metrics and structured transition modeling, but PDCorr/DE Overlap are not causal evidence.	Not reported	Not reported	[20]
CPA	unseen cell type; unseen dose; unseen combination; cross-species	Compositional perturbation representation evaluated on combinations of covariates, doses, cell contexts, and perturbations not observed jointly during training.	dose–response and model-specific baselines reported in the original study	Limited: disentangled perturbation/covariate structure is biologically interpretable but does not by itself establish causality.	Not reported	Predictive uncertainty supported in the CPA framework; reporting varies by task	[40]
Neural OT	unseen donor; unseen tissue; unseen cell state; cross-species	Population-level optimal transport evaluated on held-out biological contexts, including patient- and species-shift settings reported in the original study.	Optimal-transport and conditional generative baselines reported	Limited: transport structure encodes population transitions but causal identification depends on matching assumptions.	Not reported	Not reported	[41]
GEARS	unseen perturbation; unseen combination	Perturb-seq splits distinguish single and combinatorial perturbations with 0, 1, or 2 unseen genes in the combination; reported gains are largest for harder unseen-gene settings.	Additive and deep perturbation-prediction baselines reported	Limited: gene-graph priors and intervention data support mechanism-related interpretation, but do not establish causal mechanisms.	Not reported	Not reported	[42]
State	unseen cell type; unseen cellular context; unseen perturbation	Large perturbation datasets are split across cellular contexts and perturbations; reported evaluations emphasize transfer to held-out contexts and comparison with simple context/perturbation baselines.	Context mean and perturbation mean baselines, among others	Not evaluated	Not reported	Not reported	[21]
X-Pert	unseen cell type; unseen perturbation; unseen dose; unseen combination	Joint genetic/chemical perturbation modeling evaluated across perturbation classes and cell contexts with held-out transfer settings reported by the authors.	Task-specific perturbation baselines reported	Limited: mechanism-aligned representations/attention support biological interpretation but do not constitute causal proof.	Not reported	Not reported	[22]
Tahoe-x1	unseen cellular context; unseen perturbation	Drug-perturbation pretraining and held-out cellular-context evaluations are reported on Tahoe-scale data; performance is compared with contemporary perturbation models.	Contemporary perturbation models and simple baselines where reported	Not evaluated	Not reported	Not reported	[23]
X-Cell	unseen cell type; unseen donor; unseen perturbation	Reports zero-shot/transfer evaluations in unseen cellular backgrounds, including primary-cell and donor-shift settings, with multimodal priors used at inference.	Published perturbation-prediction baselines reported	Limited: incorporates protein, interaction, dependency, and morphology priors, but causal mechanism is not established.	Not reported	Not reported	[24]
UniPert-G2CP	cross-modal/cross-assay; unseen perturbation	Transfers phenotype knowledge from genetic perturbation screens to chemical perturbation prediction and evaluates cross-domain genetic-to-chemical generalization.	Genetic/chemical perturbation baselines reported	Limited: gene–protein–compound representations support mechanism-related transfer but do not establish full causal equivalence.	Not reported	Not reported	[45]
UCE	unseen cell state; unseen tissue; cross-species	Zero-shot cell embedding/annotation is evaluated on datasets and species not used for task-specific fine-tuning.	Representation-learning and task baselines reported	Not evaluated	Not reported	Not reported	[18]
TranscriptFormer	unseen cell state; unseen tissue; cross-species	Cross-species generative pretraining is evaluated on OOD cell-state and species transfer, including zero-shot disease/state tasks reported by the authors.	Foundation/representation baselines reported	Not evaluated	Not reported	Not reported	[19]
Nicheformer	unseen tissue; cross-platform; cross-modal/cross-assay; controlled organism-held-out transfer	Evaluated across tissues, spatial versus dissociated modalities, and controlled organism-held-out pretraining ablations. The full pretrained model includes both human and mouse data; cross-species evidence therefore refers to the controlled human-only/mouse-only ablations, not merely mixed-species pretraining.	Spatial and single-cell representation baselines reported	Not evaluated	Not reported	Confidence/uncertainty is reported for selected spatial prediction tasks; not a uniform model-wide guarantee	[6]
SpatialProp	spatial/tissue extrapolation; unseen perturbation	Spatial graph perturbation propagation is evaluated on held-out spatial perturbation settings and with CausalInteractionBench directional-interaction evaluation.	Spatial graph and perturbation baselines reported	Limited: directional-interaction benchmarking is causal-related evidence but not independent causal proof.	Not reported	Error calibration is part of the reported method; broader OOD calibration remains limited	[49]
SpaRx	cross-modal/cross-assay; spatial/tissue extrapolation	Graph-based domain adaptation transfers pharmacogenomic drug-response information to spatial single-cell transcriptomics to predict spatially heterogeneous treatment response.	Domain-adaptation and drug-response baselines reported in the original study	Not evaluated	Not reported	Not reported	[50]
SequenTx	unseen combination	A learned cellular-state/decision framework proposes sequential drug interventions that are subsequently tested experimentally; the main strength is prospective experimental validation rather than broad benchmarked OOD coverage.	Random/expert or task-specific treatment-selection comparators as reported in the study	Limited: intervention-based decision making is biologically actionable, but the study is not a full mechanistic causal model.	Demonstrated: reported *in vitro* and *in vivo* testing of model-guided treatment strategies.	Not reported	[46]

Note. Selection is based on the revision-stage Table 2 inclusion criteria plus direct relevance to prediction, transfer, perturbation response, or generalization. Selection does not depend on favorable performance, publication form, or how prominently a model was discussed in the original manuscript. A same-dataset held-out test is recorded in the evaluation column and is not treated as independent experimental validation. MVCBench [110] is treated as a benchmark/evaluation resource rather than a model row and informs the multimodal generalization discussion.

Table 7 includes predictive models selected by the same revision-stage Table 2 inclusion criteria plus direct relevance to prediction, transfer, perturbation response, or generalization. Selection does not depend on favorable results, publication form, or how extensively a model was discussed in the original manuscript.

### From directional association to causal validation

Most single-cell perturbation models lie between associational and interventional prediction. They learn conditional distributions after implemented interventions and can predict states following a specified perturbation under similar conditions, but they do not provide complete counterfactual reasoning. True counterfactuals for the same initial cell cannot be observed under both treatment and control. Population matching, latent alignment, or optimal transport therefore approximate the missing comparison [41]. Their validity depends on assumptions about unmeasured confounding, matching quality, and whether the observed population adequately represents the relevant initial states.

Directional interaction benchmarks such as CausalInteractionBench can test whether predictions align with known cell communication directions, but cannot independently confirm specific causal pathways [49].

Cross-modal transfer between genetic and chemical models can test whether perturbation representations are consistent but cannot prove a shared mechanism. Causal evaluation should consider temporal order, dose–response, intervention specificity, mediators, and replication in independent experiments.

### Unified benchmarks and community evaluation

VCBench evaluates unseen cellular backgrounds, perturbations, and merged datasets and shows that rankings depend on tasks and metrics [66]. SCMBench similarly finds that foundation models do not dominate every multi-omics integration task [65]. The Virtual Cell Challenge promotes specialized datasets and open, reproducible evaluation [82]. MVCBench extends standardized comparison to drug-induced multimodal phenotypes and explicitly evaluates representation methods under both in-distribution and OOD settings spanning unseen compounds, cell lines, assay plates, and datasets [110]. It is treated here as a benchmark and generalization-evidence resource rather than as a Table 2 virtual-cell model. Together, these efforts support reporting global agreement, perturbation-specific effects, cell-background transfer, and cross-dataset robustness.

Unified benchmarks should provide raw and normalized data, sample and cell metadata, perturbation dose and timing, negative controls, failure flags, split definitions, simple baselines, and executable evaluation code. Pretraining sources must be disclosed to identify direct or indirect overlap with test data. Results should be reported across replicates and biologically meaningful subgroups, not only as one pooled average. Application priorities differ: drug screening emphasizes false positives and uncertainty; mechanistic studies emphasize pathways and regulatory relationships; patient stratification emphasizes cross-individual transfer; and development emphasizes trajectories and morphology. Evaluation should therefore remain multidimensional rather than collapse capabilities into one score, because the same model can be credible for one use and unsupported for another.

### From prediction to actionable experimental feedback

Independent experimental validation and closed-loop experimental feedback should be distinguished. Independent experimental validation occurs when model predictions are tested in independently generated experiments. Closed-loop experimental feedback requires an additional system-level step: model predictions influence experiment selection, experimental results feed back into model updating or recalibration, and the updated information affects a subsequent decision. Independent experimental validation therefore does not by itself imply closed-loop updating.

Fully reproducible end-to-end closed-loop virtual-cell systems remain rare. SequenTx demonstrates model-guided sequential intervention design with substantial independent *in vitro* and *in vivo* experimental validation, but the reported workflow does not establish a complete experimental-feedback cycle in which new experimental observations update or recalibrate the model before a subsequent decision [46]. ProteinTalks contributes a dynamic perturbation-proteomics component that can support experimentally testable state-transition hypotheses [9], whereas Virtual Yeast is a framework-oriented Perspective describing an active experimental cycle rather than a fully validated implemented closed-loop system [54]. These examples should therefore not be interpreted as having equivalent evidentiary maturity. Evaluation of future closed-loop systems should report experimental hit rate, learning from failed experiments, information gain per round, cross-round calibration, and experimental cost savings relative to random or expert strategies.

## Challenges and outlook

### Data heterogeneity and missing negative results

Single-cell and spatial data vary across tissues, individuals, platforms, doses, and time, while coverage is much richer for transcriptomics than for perturbation proteomics, temporal epigenomics, or 3D ultrastructure [69, 70, 95]. RNA alone cannot directly represent protein abundance, phosphorylation, complex assembly, or organelle geometry [69, 92, 93, 95].

Perturbation resources also remain concentrated in common cell lines and transcriptomic endpoints. Weak, invalid, or failed perturbations are often filtered or underreported, which can bias models toward predicting meaningful effects for every intervention. Future datasets should retain negative controls, low-efficacy and failed experiments, variable delivery efficiency, and evidence that perturbations reached their intended targets.

### Out-of-distribution generalization and uncertainty quantification

Beyond appropriate benchmark design, practical deployment requires calibrated uncertainty estimates, OOD detection, and prediction-abstention mechanisms. Predictive uncertainty includes aleatoric uncertainty from measurement noise and biological stochasticity and epistemic uncertainty from incomplete training coverage. Models should output state distributions, prediction intervals, or multiple plausible trajectories rather than a single expression matrix.

For inputs far from the training distribution, models should report both OOD status and calibrated confidence and should abstain or reject predictions beyond defined applicability boundaries. Calibration should be evaluated separately across cell types, perturbation classes, doses, and platforms because a single aggregate confidence score can conceal poorly covered subgroups. These safeguards support more reliable experimental decisions and make explicit where model recommendations should not be acted upon.

### Continuous-time dynamics

Most perturbation models deal with single-step mappings between the control state and the perturbation endpoint, ignoring the intermediate temporal processes. The same endpoint may result from different dynamical pathways, and short-term stress responses may differ from long-term cellular fate. Methods such as RNA velocity use the ratio of unspliced to spliced RNA to infer the direction of local state changes, providing partial information for dynamic modeling [111]. Continuous state evolution can be described by neural ODEs [112]:


(11) \begin{eqnarray*}& \frac{dz(t)}{dt} = f_{\theta}\left( z(t),P,S(t),C,t \right).\end{eqnarray*}

When stochastic transcription and environmental fluctuations are considered, a stochastic differential equation can be used:


(12) \begin{eqnarray*}& \begin{aligned} dz(t) &= f_{\theta}\left( z(t),\xi(t),t \right)\, dt + g_{\theta}\left( z(t) \right)\, dW_{t},\\ \xi(t) &= \left( P,S(t),C \right). \end{aligned}\end{eqnarray*}

Here, $f_{\theta }$ and $g_{\theta }$ denote drift and diffusion, and $W_{t}$ is a Wiener process. State-space models, dynamic optimal transport, neural processes, and time-stamped generative models may also recover continuous trajectories. Reliable dynamics require genuine time-series data; pseudotime ordering of endpoints is not a substitute.

Continuous-time inference is additionally limited by sparse temporal sampling and the absence of one-to-one correspondence between cells at successive measurements. Snapshot populations can support multiple plausible trajectories, creating an identifiability problem that cannot be resolved by biological plausibility alone. Biological stochasticity must also be separated from measurement and model uncertainty. A biologically plausible trajectory does not by itself establish that the underlying dynamics have been correctly identified. Internal temporal generalization can be assessed with held-out time points, held-out perturbation–time combinations, or held-out trajectories where applicable. Independent temporal validation requires separately generated perturbation time series, lineage tracing, live-cell imaging, or other new temporal experiments. Held-out time points from the same dataset remain internal evaluation rather than independent validation.

### Aligning mechanistic knowledge with latent representations

Latent representations should be connected to regulatory networks, signaling pathways, metabolic reactions, and organelle functions rather than interpreted from attention alone. Regulatory modules, graph priors, conservation relations, and reaction constraints can provide intermediate biological structure [7, 16, 29], but their value must be tested by interventions rather than reconstruction quality or plausible attention maps. Because prior knowledge is incomplete and context-dependent, known networks are better treated as updatable soft constraints than fixed truth.

### Joint cross-scale modeling

Linking molecular expression to cellular phenotype and tissue structure remains difficult because single-cell, agent-based, and tissue-mechanics models use different variables and timescales [52–54, 71]. The following conceptual framework abstracts cross-scale transfer and is not a specific existing model. For simplicity, population effects are included in tissue aggregation; an explicit population state may be inserted when required:


(13) \begin{eqnarray*} & z_{t}^{mol} = E_{mol}\left( X_{t} \right).\end{eqnarray*}

(14) \begin{eqnarray*}& z_{t}^{cell} = E_{cell}\left( z_{t}^{mol},M_{t} \right).\end{eqnarray*}

(15) \begin{eqnarray*}& z_{t}^{tissue} = E_{tissue}\left( \{ z_{t,i}^{cell}\}_{i = 1}^{N},S_{t} \right).\end{eqnarray*}

Here, $z_{t}^{mol}$, $z_{t}^{cell}$, and $z_{t}^{tissue}$ are molecular, cellular, and tissue latent states; $z_{t,i}^{cell}$ is the state of cell *i*; *N* is the number of cells; and $S_{t}$ is spatial and tissue context. Ultrastructure can be included in $M_{t}$ or modeled as an intermediate layer. A hierarchical model should propagate information upward and allow tissue context to regulate lower-level states.

Concrete cross-scale interfaces are required for this hierarchy to become operational. Molecular-to-cell mappings may translate pathway activity, protein abundance, metabolic state, receptor or signaling activity, and cell-cycle state into probabilities or rates of proliferation, death, differentiation, migration, and secretion. Cell-to-tissue interfaces may aggregate cell density, interaction forces, ligand fields, extracellular matrix state, and spatial neighborhoods. Tissue-level feedback can in turn modify cellular states through hypoxia, nutrients, mechanical stress, cytokines, and cell–cell contact. Because these processes evolve on different timescales, implementations may require multirate updating, event-based synchronization, or explicit coarse/fine temporal alignment. Validation must therefore test not only outputs at each scale but also the cross-scale mapping itself.

Cross-scale models should be validated layer by layer: transcriptomics and proteomics at the molecular level, morphology and fate tracking at the cellular level, and spatial omics and imaging at the tissue level. The mapping between levels also requires validation. Correct molecular predictions do not guarantee correct cell behavior, and correct local cell states do not guarantee tissue morphology. Simultaneous agreement across levels, preferably under the same intervention and over time, provides stronger evidence for reliable inter-level relationships.

### Active experimental design and sequential learning

Virtual cells should improve experimental design rather than replace experiments. A simple acquisition objective can balance expected information gain against experimental cost [1, 46, 70]:


(16) \begin{eqnarray*}& P^{*} = \arg\max_{P}\{I(P)-\lambda K(P)\}.\end{eqnarray*}

Selection should favor perturbations that distinguish mechanisms or reduce uncertainty, not merely those predicted to have large effects. Sequential learning also requires records of data versions, model configuration, confidence, rejected recommendations, failed experiments, and what changed between rounds. Drug-screening or precision-medicine claims should retain a validation ladder from computational prediction to appropriate *in vitro*, organoid, animal, or clinical testing rather than equating expression prediction with therapeutic efficacy.

### Open standards and reporting guidelines

Virtual-cell studies remain difficult to compare because data formats, preprocessing, perturbation encoding, and evaluation splits differ. Reports should specify state variables, spatial context, perturbation, dose, time, uncertainty, preprocessing, baselines, code/weights where possible, and applicability limits. Rapidly updated models and preprints should additionally record publication form, versioned model cards, training-corpus or weight changes, and benchmark versions so that later evaluations can be tied to the exact artifact tested.

## Conclusion

Virtual-cell research is moving from transcriptome-centered representation toward multimodal, perturbation-responsive, spatial, and cross-scale systems. In this review, E/T/D is a conceptual comparative abstraction and LCM/AIVC is an operational analytical distinction, not a mandatory architecture or field-wide taxonomy. Evidence is assessed across complementary DOC, TASK, GEN, MECH, and EXP dimensions, with closed-loop experimental feedback reported separately.

Progress depends less on parameter scale or publication form than on reproducible resources, explicit distribution-shift evaluation, mechanistic or causal support where claimed, and independent experiments. RNA alone cannot represent functional, spatial, and ultrastructural state, and cross-scale models must validate the mappings that connect molecular, cellular, and tissue variables. The most trustworthy virtual cells will therefore combine multimodal state transitions, calibrated uncertainty, mechanistic constraints, and experimentally testable predictions while making their applicability boundaries explicit [5, 9, 11–14, 25, 26, 53, 64–66].

**Key Points**

- Model scale alone does not define a virtual cell; perturbation-responsive transitions and experimentally testable outputs are separate requirements.
- E/T/D is a conceptual comparative abstraction rather than a mandatory architectural decomposition, and the LCM/AIVC distinction is operational.
- Virtual-cell evidence should be assessed across distinct generalization, mechanistic/causal, and experimental dimensions rather than inferred from model scale or publication form.
- Transcriptomic states are insufficient alone; multimodal, spatial, multiscale, and ultrastructural layers require explicit alignment and validation.
- Closed-loop experimental feedback remains an emerging system-level objective and should be distinguished from independent experimental validation.

## Supplementary Material

Virtual_Cells_Supplementary_Materials_REVISED_bbag562

## Data Availability

No new experimental data were generated for this review. The publications, repositories, and resources discussed are identified in the reference list and tables.

## References

[1] Bunne C, Roohani Y, Rosen Y et al. How to build the virtual cell with artificial intelligence: priorities and opportunities. *Cell* 2024;187:7045–63. 10.1016/j.cell.2024.11.015 PMC12148494 39672099

[2] Loew LM, Schaff JC. The virtual cell: a software environment for computational cell biology. *Trends Biotechnol* 2001;19:401–6. 10.1016/s0167-7799(01)01740-1 11587765

[3] Ghaffarizadeh A, Heiland R, Friedman SH et al. PhysiCell: an open source physics-based cell simulator for 3-D multicellular systems. *PLoS Comput Biol* 2018;14:e1005991. 10.1371/journal.pcbi.1005991 PMC5841829 29474446

[4] Karr JR, Sanghvi JC, Macklin DN et al. A whole-cell computational model predicts phenotype from genotype. *Cell* 2012;150:389–401. 10.1016/j.cell.2012.05.044 PMC3413483 22817898

[5] Chen X, Li K, Cui X et al. EpiAgent: foundation model for single-cell epigenomics. *Nat Methods* 2025;22:2316–27. 10.1038/s41592-025-02822-z 40999099

[6] Tejada-Lapuerta A, Schaar AC, Gutgesell R et al. Nicheformer: a foundation model for single-cell and spatial omics. *Nat Methods* 2025;22:2525–38. 10.1038/s41592-025-02814-z PMC12695652 41168487

[7] Hu L, Qin H, Zhang Y et al. RegFormer: a single-cell foundation model powered by gene regulatory hierarchies. *Nat Commun* 2026;17:6432. 10.1038/s41467-026-72198-x PMC13376892 42086551

[8] Jiang Q, Li Z, Hu B et al. HoloCell: a generative foundation model for holistic cellular modeling. bioRxiv. 2026. 10.64898/2026.06.07.730684

[9] Sun R, Qian L, Li Y et al. An operational perturbation proteomics-based virtual cell model. *Nature* 2026. 10.1038/s41586-026-11001-9 42717098

[10] Zhao S, Luo Y, Yang G et al. SToFM: a multi-scale foundation model for spatial transcriptomics. In: Proceedings of the 42nd international conference on machine learning. *PMLR* 2025;267:77589–607. https://proceedings.mlr.press/v267/zhao25p.html

[11] He L, Shi R, Wang W et al. *Unifying the electron microscopy multiverse through a large-scale foundation model*. bioRxiv. 2025. 10.1101/2025.04.13.648639

[12] Yu Z, Guo J, Liu F et al. EMCF ecosystem: towards pretrained foundation model for electron microscopy image analysis. bioRxiv. 2025. 10.64898/2025.12.09.693109

[13] Du M, Wang Y, Xie L et al. A foundation AI model enhances electron microscopy image analysis. bioRxiv. 2026. 10.64898/2026.02.28.708664

[14] Ji B, Hu T, Wang J et al. CAPTAIN: a multimodal foundation model pretrained on co-assayed single-cell RNA and protein. *Nat Commun* 2026;17:6161. 10.1038/s41467-026-72882-y PMC13365403 42098152

[15] Cui H, Wang C, Maan H et al. scGPT: toward building a foundation model for single-cell multi-omics using generative AI. *Nat Methods* 2024;21:1470–80. 10.1038/s41592-024-02201-0 38409223

[16] Theodoris CV, Xiao L, Chopra A et al. Transfer learning enables predictions in network biology. *Nature* 2023;618:616–24. 10.1038/s41586-023-06139-9 PMC10949956 37258680

[17] Hao M, Gong J, Zeng X et al. Large-scale foundation model on single-cell transcriptomics. *Nat Methods* 2024;21:1481–91. 10.1038/s41592-024-02305-7 38844628

[18] Rosen Y, Roohani Y, Agrawal A et al. Universal cell embedding provides a foundation model for cell biology. *Nature* 2026;656:183–91. 10.1038/s41586-026-10689-z PMC13441871 42420460

[19] Pearce JD, Simmonds SE, Mahmoudabadi G et al. TranscriptFormer: a generative cell atlas across 1.5 billion years of evolution. *Science* 2026;393:aec8514. 10.1126/science.aec8514 42096520

[20] Chen S, Yu L, Lin X et al. SCALE: scalable conditional atlas-level endpoint transport for virtual cell perturbation prediction. bioRxiv. 2026. 10.64898/2026.03.17.712536

[21] Adduri AK, Gautam D, Bevilacqua B et al. Predicting cellular responses to perturbation across diverse contexts with state. *Cell* 2026;189:5914–5931.e20. 10.1016/j.cell.2026.07.052 42673963

[22] Li C, Wei L, Zhang X. *Unified multimodal learning enables generalized cellular response prediction to diverse perturbations*. bioRxiv. 2025. 10.1101/2025.11.13.688367

[23] Gandhi S, Javadi F, Svensson V et al. Tahoe-x1: scaling perturbation-trained single-cell foundation models to 3 billion parameters. bioRxiv. 2025. 10.1101/2025.10.23.683759

[24] Wang C, Karimzadeh M, Ravindra NG et al. X-cell: scaling causal perturbation prediction across diverse cellular contexts via diffusion language models. bioRxiv. 2026. 10.64898/2026.03.18.712807

[25] Ahlmann-Eltze C, Huber W, Anders S. Deep-learning-based gene perturbation effect prediction does not yet outperform simple linear baselines. *Nat Methods* 2025;22:1657–61. 10.1038/s41592-025-02772-6 PMC12328236 40759747

[26] Kedzierska KZ, Crawford L, Amini AP et al. Zero-shot evaluation reveals limitations of single-cell foundation models. *Genome Biol* 2025;26:101. 10.1186/s13059-025-03574-x PMC12007350 40251685

[27] Yang F, Wang W, Wang F et al. scBERT as a large-scale pretrained deep language model for cell type annotation of single-cell RNA-seq data. *Nat Mach Intell* 2022;4:852–66. 10.1038/s42256-022-00534-z

[28] Wen H, Tang W, Dai X et al. CellPLM: pre-training of cell language model beyond single cells. In: Kim B, Yue Y, Chaudhuri S, Fragkiadaki K, Khan M, Sun Y (eds.), International Conference on Learning Representations. Appleton, WI: International Conference on Learning Representations, 2024. https://openreview.net/forum?id=BKXvPDekud (8 August 2026, date last accessed).

[29] Yang X, Liu G, Feng G et al. GeneCompass: deciphering universal gene regulatory mechanisms with a knowledge-informed cross-species foundation model. *Cell Res* 2024;34:830–45. 10.1038/s41422-024-01034-y PMC11615217 39375485

[30] Bian H, Chen Y, Dong X et al. scMulan: a multitask generative pre-trained language model for single-cell analysis. In: Ma J (ed.), Research in Computational Molecular Biology, Lecture Notes in Computer Science, Vol. 14758. Cham: Springer, 2024, 479–82. 10.1007/978-1-0716-3989-4_57

[31] Levine D, Rizvi SA, Lévy S et al. Cell2Sentence: teaching large language models the language of biology. In: Proceedings of the 41st international conference on machine learning. *PMLR* 2024;235:27299–325. https://proceedings.mlr.press/v235/levine24a.html

[32] Jiang X, Xie J. A transcriptomics-native foundation model for universal cell representation and virtual cell synthesis. bioRxiv. 2026. 10.64898/2026.04.12.718016

[33] Li G, Fu S, Wang S et al. A deep generative model for multi-view profiling of single-cell RNA-seq and ATAC-seq data. *Genome Biol* 2022;23:20. 10.1186/s13059-021-02595-6 PMC8756637 35022082

[34] Xiong L, Chen T, Kellis M. scCLIP: multi-modal single-cell contrastive learning integration pre-training. *NeurIPS AI for Science Workshop* 2023. https://openreview.net/forum?id=KMtM5ZHxct (8 August 2026, date last accessed)

[35] Xu J, Huang DS, Zhang X. scmFormer integrates large-scale single-cell proteomics and transcriptomics data by multi-task transformer. *Adv Sci (Weinh)* 2024;11:2307835. 10.1002/advs.202307835 PMC11109621 38483032

[36] Wu J, Wan C, Ji Z et al. *EpiFoundation: a foundation model for single-cell ATAC-seq via peak-to-gene alignment*. bioRxiv. 2025. 10.1101/2025.02.05.636688

[37] Gao Y, Wang W, Zhao Y et al. Language may be all omics needs: harmonizing multimodal data for omics understanding with CellHermes. bioRxiv. 2025. 10.1101/2025.11.07.687322

[38] Li B, Liu Z, Wang Z et al. CLM-X: a multimodal single-cell foundation model with flexible multi-way transformer for unified scRNA-seq and scATAC-seq analysis. bioRxiv. 2026. 10.64898/2026.02.17.704943

[39] Liu G, Zhao Y, Zhao Y et al. SCARF: single cell ATAC-seq and RNA-seq foundation model. bioRxiv. 2025. 10.1101/2025.04.07.647689

[40] Lotfollahi M, Klimovskaia Susmelj A, De Donno C et al. Predicting cellular responses to complex perturbations in high-throughput screens. *Mol Syst Biol* 2023;19:e11517. 10.15252/msb.202211517 PMC10258562 37154091

[41] Bunne C, Stark SG, Gut G et al. Learning single-cell perturbation responses using neural optimal transport. *Nat Methods* 2023;20:1759–68. 10.1038/s41592-023-01969-x PMC10630137 37770709

[42] Roohani Y, Huang K, Leskovec J. Predicting transcriptional outcomes of novel multigene perturbations with GEARS. *Nat Biotechnol* 2024;42:927–35. 10.1038/s41587-023-01905-6 PMC11180609 37592036

[43] Dong M, Adduri A, Gautam D et al. Stack: in-context learning of single-cell biology. bioRxiv. 2026. 10.64898/2026.01.09.698608

[44] Chuai G, Chen X, Yang X et al. Towards building a world model to simulate perturbation-induced cellular dynamics by AlphaCell. bioRxiv. 2026. 10.64898/2026.03.02.709176

[45] Li Y, Zeng M, Zhu J et al. UniPert-G2CP bridges genetic and chemical screens from molecular representation to phenotype modeling. *Cell* 2026;189:4946–4963.e8. 10.1016/j.cell.2026.06.005 42497867

[46] Chen X, Deng Y, Yang X et al. Reinforcement learning-based design of sequential drug treatment targeting the evolving tumour landscape with SequenTx. *Nat Mach Intell* 2026;8:351–71. 10.1038/s42256-026-01192-1

[47] Birk S, Bonafonte-Pardàs I, Feriz AM et al. Quantitative characterization of cell niches in spatially resolved omics data. *Nat Genet* 2025;57:897–909. 10.1038/s41588-025-02120-6 PMC11985353 40102688

[48] Hu L, Zhang S, Zhang X et al. *STARNet enables spatially resolved inference of gene regulatory networks from spatial multi-omics data*. bioRxiv. 2025. 10.1101/2025.08.21.671434

[49] Sun ED, Buendia A, Brunet A et al. SpatialProp: tissue perturbation modeling with spatially resolved single-cell transcriptomics. bioRxiv. 2025. 10.64898/2025.11.30.691355

[50] Tang Z, Liu X, Li Z et al. SpaRx: elucidate single-cell spatial heterogeneity of drug responses for personalized treatment. *Brief Bioinform* 2023;24:bbad338. 10.1093/bib/bbad338 PMC10555713 37798249

[51] Kalafut NC, He C, Sheng J et al. Inferring virtual cell environments using multi-agent reinforcement learning. bioRxiv. 2025. 10.1101/2025.11.21.689815

[52] Cao N, Lu Y, Qiu X. Towards predictive virtual embryos with genomics and AI. *Nat Methods* 2026;23:1666–70. 10.1038/s41592-026-03055-4 41888299

[53] Wenckstern J, Jain E, von Querfurth B et al. The virtual tissues foundation model resolves spatial proteomics across scales. *Nature* 2026;657:744–54. 10.1038/s41586-026-10884-y PMC13581600 42557331

[54] Qian L, Zhou Z, Zhou P et al. Towards the construction of a virtual yeast. *Nature* 2026;655:59–70. 10.1038/s41586-026-10574-9 42387167

[55] Jiang Q, Ye X, Guo Z et al. Phenotype-guided in silico molecular generation using large language models. bioRxiv. 2026. 10.64898/2026.01.03.697483

[56] Zhou Q, Le Y, Qi X et al. CellOS: learning a world model of cellular state through joint embedding prediction. bioRxiv. 2026. 10.64898/2026.06.18.733163

[57] Harris LA, Hogg JS, Tapia JJ et al. BioNetGen 2.2: advances in rule-based modeling. *Bioinformatics* 2016;32:3366–8. 10.1093/bioinformatics/btw469 PMC5079481 27402907

[58] Swat MH, Thomas GL, Belmonte JM et al. Multi-scale modeling of tissues using CompuCell3D. *Methods Cell Biol* 2012;110:325–66. 10.1016/B978-0-12-388403-9.00013-8 PMC3612985 22482955

[59] Starruß J, de Back W, Brusch L et al. Morpheus: a user-friendly modeling environment for multiscale and multicellular systems biology. *Bioinformatics* 2014;30:1331–2. 10.1093/bioinformatics/btt772 PMC3998129 24443380

[60] Baek S, Song K, Lee I. Single-cell foundation models: bringing artificial intelligence into cell biology. *Exp Mol Med* 2025;57:2169–81. 10.1038/s12276-025-01547-5 PMC12586647 41028523

[61] Yiu T, Chen B, Wang H et al. Transformative advances in single-cell omics: a comprehensive review of foundation models, multimodal integration and computational ecosystems. *J Transl Med* 2025;23:1176. 10.1186/s12967-025-07091-0 PMC12560279 41146276

[62] Valanarasu JMJ, Xu H, Usuyama N et al. Multimodal AI generates virtual population for tumor microenvironment modeling. *Cell* 2026;189:386–400.e19. 10.1016/j.cell.2025.11.016 41371214

[63] Dimitrov D, Schrod S, Rohbeck M et al. Interpretation, extrapolation and perturbation of single cells. *Nat Rev Genet* 2026;27:349–70. 10.1038/s41576-025-00920-4 41478891

[64] DenAdel A, Hughes M, Thoutam A et al. Evaluating the role of pretraining dataset size and diversity on single-cell foundation model performance. *Nat Methods* 2026;23:1447–57. 10.1038/s41592-026-03120-y PMC13412022 42265208

[65] Wang Y, Fan Y, Wang X et al. SCMBench: benchmarking domain-specific and foundation models for single-cell multi-omics data integration. *Nat Commun* 2026;17:5967. 10.1038/s41467-026-72570-x PMC13342640 42069651

[66] Mao X, Zhang S, Wen Q et al. Benchmarking virtual cell models for in-the-wild perturbation response. arXiv:2604.27646. 2026. https://arxiv.org/abs/2604.27646 (8 August 2026, date last accessed)

[67] Hao M, Wei L, Yang F et al. Current opinions on large cellular models. *Quant Biol* 2024;12:433–43. 10.1002/qub2.65 PMC12805985 41674875

[68] Ma C, Zhang H, Rao Y et al. AI-driven virtual cell models in preclinical research: technical pathways, validation mechanisms, and clinical translation potential. *NPJ Digit Med* 2026;9:25. 10.1038/s41746-025-02198-6 PMC12789685 41381900

[69] Sun Y, J A, Liu Z et al. AI proteomics: from protein identification to virtual cells. *Nat Methods* 2026;23:1697–709. 10.1038/s41592-026-03085-y 42521824

[70] Qian L, Dong Z, Guo T. Grow AI virtual cells: three data pillars and closed-loop learning. *Cell Res* 2025;35:319–21. 10.1038/s41422-025-01101-y PMC12012092 40128605

[71] Cui H, Tejada-Lapuerta A, Brbić M et al. Towards multimodal foundation models in molecular cell biology. *Nature* 2025;640:623–33. 10.1038/s41586-025-08710-y 40240854

[72] Youngblut ND, Carpenter C, Nayebnazar A et al. scBaseCount: an AI agent-curated, standardized, auto-updated single-cell data repository. Cell 2026;189:5932–44.e6. 10.1016/j.cell.2026.08.025 42753696

[73] [dataset] Arc Institute . Arc virtual cell atlas. 2026. https://arcinstitute.org/tools/virtualcellatlas (8 August 2026, date last accessed).

[74] [dataset] Chan Zuckerberg Initiative . CELLxGENE discover and census. 2026. https://cellxgene.cziscience.com (8 August 2026, date last accessed).

[75] Tabula Sapiens Consortium . The tabula sapiens: a multiple-organ, single-cell transcriptomic atlas of humans. *Science* 2022;376:eabl4896. 10.1126/science.abl4896 PMC9812260 35549404

[76] HuBMAP Consortium . The human body at cellular resolution: the NIH human biomolecular atlas program. *Nature* 2019;574:187–92. 10.1038/s41586-019-1629-x PMC6800388 31597973

[77] Liu J, Lichtenberg T, Hoadley KA et al. An integrated TCGA pan-cancer clinical data resource to drive high-quality survival outcome analytics. *Cell* 2018;173:400–416.e11. 10.1016/j.cell.2018.02.052 PMC6066282 29625055

[78] Ghandi M, Huang FW, Jané-Valbuena J et al. Next-generation characterization of the Cancer Cell Line Encyclopedia. *Nature* 2019;569:503–8. 10.1038/s41586-019-1186-3 PMC6697103 31068700

[79] Srivatsan SR, McFaline-Figueroa JL, Ramani V et al. Massively multiplex chemical transcriptomics at single-cell resolution. *Science* 2020;367:45–51. 10.1126/science.aax6234 PMC7289078 31806696

[80] [dataset] Parse Biosciences . 10 million human PBMCs from a 12-donor cohort with 90 cytokine stimulations. 2024. https://www.parsebiosciences.com/datasets/10-million-human-pbmcs-in-a-single-experiment/ (8 August 2026, date last accessed).

[81] Subramanian A, Narayan R, Corsello SM et al. A next generation connectivity map: L1000 platform and the first 1,000,000 profiles. *Cell* 2017;171:1437–1452.e17. 10.1016/j.cell.2017.10.049 PMC5990023 29195078

[82] Roohani YH, Hua TJ, Tung PY et al. Virtual cell challenge: toward a Turing test for the virtual cell. *Cell* 2025;188:3370–4. 10.1016/j.cell.2025.06.008 40578317

[83] Arc Institute . Behind the data of the virtual cell challenge. 2025. https://arcinstitute.org/news/behind-the-data-virtual-cell-challenge (8 August 2026, date last accessed).

[84] Replogle JM, Saunders RA, Pogson AN et al. Mapping information-rich genotype-phenotype landscapes with genome-scale perturb-seq. *Cell* 2022;185:2559–2575.e28. 10.1016/j.cell.2022.05.013 PMC9380471 35688146

[85] Adamson B, Norman TM, Jost M et al. A multiplexed single-cell CRISPR screening platform enables systematic dissection of the unfolded protein response. *Cell* 2016;167:1867–1882.e21. 10.1016/j.cell.2016.11.048 PMC5315571 27984733

[86] Dixit A, Parnas O, Li B et al. Perturb-seq: dissecting molecular circuits with scalable single-cell RNA profiling of pooled genetic screens. *Cell* 2016;167:1853–1866.e17. 10.1016/j.cell.2016.11.038 PMC5181115 27984732

[87] Norman TM, Horlbeck MA, Replogle JM et al. Exploring genetic interaction manifolds constructed from rich single-cell phenotypes. *Science* 2019;365:786–93. 10.1126/science.aax4438 PMC6746554 31395745

[88] [dataset] National Center for Biotechnology Information . GSE264667: transcriptome-wide characterization of genetic perturbations. 2025. https://www.ncbi.nlm.nih.gov/geo/query/acc.cgi?acc=GSE264667 (8 August 2026, date last accessed).

[89] [dataset] National Center for Biotechnology Information . GSE281048: systematic reconstruction of molecular pathway signatures using scalable single-cell perturbation screens. 2025. https://www.ncbi.nlm.nih.gov/geo/query/acc.cgi?acc=GSE281048 (8 August 2026, date last accessed). 10.1038/s41556-025-01622-z PMC12083445 40011560

[90] [dataset] Huang AC, Chu C. Processed data for X-atlas/Orion: genome-wide perturb-seq datasets via a scalable fix-cryopreserve platform for training dose-dependent biological foundation models. *Figshare* 2025. 10.25452/figshare.plus.29190726

[91] Peidli S, Green TD, Shen C et al. scPerturb: harmonized single-cell perturbation data. *Nat Methods* 2024;21:531–40. 10.1038/s41592-023-02144-y PMC12220817 38279009

[92] Thul PJ, Åkesson L, Wiking M et al. A subcellular map of the human proteome. *Science* 2017;356:eaal3321. 10.1126/science.aal3321 28495876

[93] Cho NH, Cheveralls KC, Brunner AD et al. OpenCell: endogenous tagging for the cartography of human cellular organization. *Science* 2022;375:eabi6983. 10.1126/science.abi6983 PMC9119736 35271311

[94] Chandrasekaran SN, Cimini BA, Goodale A et al. Three million images and morphological profiles of cells treated with matched chemical and genetic perturbations. *Nat Methods* 2024;21:1114–21. 10.1038/s41592-024-02241-6 PMC11166567 38594452

[95] Heinrich L, Bennett D, Ackerman D et al. Whole-cell organelle segmentation in volume electron microscopy. *Nature* 2021;599:141–6. 10.1038/s41586-021-03977-3 34616042

[96] Iorio F, Knijnenburg TA, Vis DJ et al. A landscape of pharmacogenomic interactions in cancer. *Cell* 2016;166:740–54. 10.1016/j.cell.2016.06.017 PMC4967469 27397505

[97] Corsello SM, Nagari RT, Spangler RD et al. Discovering the anticancer potential of non-oncology drugs by systematic viability profiling. *Nat Cancer* 2020;1:235–48. 10.1038/s43018-019-0018-6 PMC7328899 32613204

[98] Zagidullin B, Aldahdooh J, Zheng S et al. DrugComb: an integrative cancer drug combination data portal. *Nucleic Acids Res* 2019;47:W43–51. 10.1093/nar/gkz337 PMC6602441 31066443

[99] Nadal-Ribelles M, Solé C, Díez-Villanueva A et al. A single-cell resolved genotype-phenotype map using genome-wide genetic and environmental perturbations. *Nat Commun* 2025;16:2645. 10.1038/s41467-025-57600-4 PMC11920212 40102404

[100] UniProt Consortium . UniProt: the universal protein knowledgebase in 2025. *Nucleic Acids Res* 2025;53:D609–17. 10.1093/nar/gkae1010 PMC11701636 39552041

[101] Gene Ontology Consortium . The gene ontology knowledgebase in 2023. *Genetics* 2023;224:iyad031. 10.1093/genetics/iyad031 PMC10158837 36866529

[102] Milacic M, Beavers D, Conley P et al. The Reactome Pathway Knowledgebase 2024. *Nucleic Acids Res* 2024;52:D672–8. 10.1093/nar/gkad1025 PMC10767911 37941124

[103] Szklarczyk D, Kirsch R, Koutrouli M et al. The STRING database in 2023: protein–protein association networks and functional enrichment analyses for any sequenced genome of interest. *Nucleic Acids Res* 2023;51:D638–46. 10.1093/nar/gkac1000 PMC9825434 36370105

[104] Giurgiu M, Reinhard J, Brauner B et al. CORUM: the comprehensive resource of mammalian protein complexes, 2019. *Nucleic Acids Res* 2019;47:D559–63. 10.1093/nar/gky973 PMC6323970 30357367

[105] Tweedie S, Braschi B, Gray K et al. Genenames.Org: the HGNC and VGNC resources in 2021. *Nucleic Acids Res* 2021;49:D939–46. 10.1093/nar/gkaa980 PMC7779007 33152070

[106] Mudge JM, Carbonell-Sala S, Diekhans M et al. GENCODE 2025: reference gene annotation for human and mouse. *Nucleic Acids Res* 2025;53:D966–75. 10.1093/nar/gkae1078 PMC11701607 39565199

[107] European Molecular Biology Laboratory . Ensembl BioMart. 2026. https://mart.ensembl.org (8 August 2026, date last accessed).

[108] Cherry JM, Hong EL, Amundsen C et al. Saccharomyces genome database: the genomics resource of budding yeast. *Nucleic Acids Res* 2012;40:D700–5. 10.1093/nar/gkr1029 PMC3245034 22110037

[109] Lab GENTEL . VCWorld and GeneTAK benchmark. 2026. https://github.com/GENTEL-lab/VCWorld (8 August 2026, date last accessed).

[110] Li B, Wang Q, Wang S et al. MVCBench: a multimodal benchmark for drug-induced virtual cell phenotypes. bioRxiv. 2026. 10.64898/2026.04.22.720110

[111] Bergen V, Lange M, Peidli S et al. Generalizing RNA velocity to transient cell states through dynamical modeling. *Nat Biotechnol* 2020;38:1408–14. 10.1038/s41587-020-0591-3 32747759

[112] Chen RTQ, Rubanova Y, Bettencourt J et al. Neural ordinary differential equations. In: Bengio S, Wallach H, Larochelle H, Grauman K, Cesa-Bianchi N, Garnett R (eds.), *Advances in Neural Information Processing Systems*, Vol. 31, pp. 6571–83. Red Hook, NY: Curran Associates, 2018. https://proceedings.neurips.cc/paper/2018/hash/69386f6bb1dfed68692a24c8686939b9-Abstract.html

